# Predicting Coral Cover Trends From Local to Broad Spatial Scales

**DOI:** 10.1111/gcb.70998

**Published:** 2026-08-05

**Authors:** Julie Vercelloni, Murray Logan, Andrew Zammit‐Mangion, Matthew Sainsbury‐Dale, Britta Schaffelke, Kerrie Mengersen, Manuel González‐Rivero

**Affiliations:** ^1^ Australian Institute of Marine Science Townsville MC Queensland Australia; ^2^ Centre for Data Science Queensland University of Technology Brisbane Queensland Australia; ^3^ School of Mathematics and Statistics University of New South Wales Sydney New South Wales Australia; ^4^ Securing Antarctica's Environmental Future University of Wollongong Wollongong New South Wales Australia; ^5^ Statistics Program, Computer, Electrical and Mathematical Sciences and Engineering Division King Abdullah University of Science and Technology Thuwal Saudi Arabia; ^6^ School of Mathematical Sciences Queensland University of Technology Brisbane Queensland Australia

**Keywords:** Bayesian, coral reefs, disturbances, long‐term data, monitoring, predictive model, spatio‐temporal dependence structure, uncertainty

## Abstract

Modern biodiversity monitoring programs are designed to provide rapid assessments of trends in the abundance and distribution of keystone taxa and timely scientific insights for decision‐making. An important consideration when delivering this information to stakeholders is the quantification of uncertainty, which determines the robustness of detecting changes across habitats and regions. In coral reef science, sparse and fragmented monitoring datasets hinder assessments of reef habitat changes. We introduce a comprehensive prediction framework for estimating trends in hard coral cover and associated uncertainty at a local scale (5 km^2^ predictive cells). The model accounts for spatial and temporal dependencies in coral cover through a latent process formulation, where correlation is structured explicitly in space and time, and includes environmental variables describing exposure to heat stress and tropical cyclones. We use a weighted spatial aggregation approach to predict trends at subregional and regional scales, with subregional and regional extents varying according to geographic context. The same approach is applied to estimate trends at broader spatial scales by combining outputs from multiple models through the ReefCloud platform, ensuring that predictions reflect the spatial distribution of reef‐building corals and that uncertainty is appropriately propagated across spatial scales. The model also quantifies effects of heat stress and tropical cyclones and characterizes their associations with coral cover change across gradients of disturbance intensity. We demonstrate the value of the framework using two use cases: the central Great Barrier Reef in Australia and American Samoa. Together, these applications highlight the potential of our integrated approach as a widely applicable method for predicting coral cover trends at various spatial scales. We also discuss the substantial uncertainty associated with the framework due to the limitations of available datasets and suggest approaches to improve the robustness of trend detection for coral reefs and their attribution to environmental disturbances.

## Graphical abstract

We present an integrated framework to track coral cover trends across multiple spatial scales, ranging from a local scale of 5 km^2^ to a broad scale exceeding 5000 km^2^. Through use cases, we demonstrate the value of our spatio‐temporal prediction framework in generating new insights from monitoring data across varying spatial and temporal data resolutions, by estimating coral cover trends at scales relevant to regional decision making, attributing changes to environmental disturbances and quantifying the influence of uncertainty on the robustness of trend detection. These applications highlight the potential of our integrated approach as a widely applicable method for predicting coral cover trends from local to broad spatial scales.

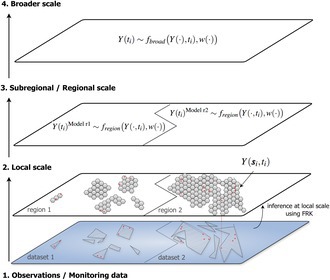

At the heart of addressing the biodiversity crisis, global efforts focus on obtaining reliable trends in abundance and distribution across taxonomic groups and biodiversity metrics, while explicitly accounting for dependence structure in monitoring data (Dornelas et al. 2023; Johnson et al. 2024). Used as an indicator, a trend change in the abundance or distribution of keystone taxa supports management plans and policy interventions to maintain biodiversity and ecosystem functions (Lindenmayer and Likens 2010; Tekwa et al. 2023; Gonzalez et al. 2023; Johnson et al. 2024). A key challenge is the robust detection of increasing and decreasing trends in the presence of sparse monitoring data, sampling biases, and limitations in statistical approaches to account for uncertainty and multiple non‐independent correlation structures, including those in space and time (Thomas 1996; Yoccoz et al. 2001; Peterson et al. 2020; Hughes et al. 2021; Dornelas et al. 2023; Tekwa et al. 2023; Johnson et al. 2024; Boënnec et al. 2024). Additional monitoring data can alleviate some of the above challenges by improving the confidence in trend detection and enabling more effective data‐driven management actions (Leung and Gonzalez 2024), and forecasting (Dietze et al. 2018; Edmunds 2024). However, the scale and speed of habitat deterioration is sometimes so substantial that simply increasing the volume of monitoring data is unlikely to be sufficient to improve change detection, which remains constrained by cost and time (Yoccoz et al. 2001; King and Halpern 2025). Methodologies that can extract deeper insights from existing datasets, represent complex dependence structure, improve interpretability, and explicitly account for uncertainty are urgently needed (Wikle et al. 2019).

Automating biodiversity assessments is essential for providing timely evidence to stakeholders (Keitt and Abelson 2021). The Global Biodiversity Information Facility (GBIF) exemplifies how digital platform engineering through the automation of data processing workflows and the integration of diverse data sources provides greater global coverage (Moritz et al. 2011). Open data also supports international biodiversity frameworks such as the Kunming‐Montreal Global Biodiversity Framework and drives advances in the detection of population trend changes (Dornelas et al. 2023; Leung and Gonzalez 2024; Johnson et al. 2024). Despite an increasing number of digital platforms across regions, additional work is needed to address persistent geographic and taxonomic gaps (Caldwell et al. 2024; King and Halpern 2025), particularly in biodiversity‐rich countries (Stephenson 2020).

Marine ecosystems present additional challenges for monitoring and assessing habitat conditions due to the limitations of traditional survey methods, which are often costly and time‐consuming (Ditria et al. 2022; Borja et al. 2024). As a result, monitoring efforts are not necessarily concentrated in countries with the most unique marine biodiversity or the greatest environmental threats, but rather in those with higher per capita gross domestic product, where wealthier nations have a greater capacity to support long‐term monitoring programs (Fisher et al. 2011; McClanahan and Rankin 2016; Moussy et al. 2022). Across the world, a major constraint in tracking changes in the condition of coral reef ecosystems is the limited availability of long‐term monitoring data (i.e., more than 15 years of sampling) due to irregular data collection, or sampling at time intervals not capturing the complex responses of Scleractinian hard corals to environmental pressures (Obura et al. 2019; Peterson et al. 2020; Mellin et al. 2020; Souter et al. 2021). For example, despite representing 26% of the global coral reef area and being highly susceptible to climate change impacts, including marine heat stress and tropical cyclones, only 50 locations in the Pacific region of the Global Coral Reef Monitoring Network (GCRMN) are regularly sampled (Souter et al. 2021; Wicquart et al. 2025). By comparison, in the Australian GCRMN region, 157 monitored locations are classified as long‐term, of which 89.8% are situated along the Great Barrier Reef (GBR) (Souter et al. 2021). Many more reef locations are surveyed in these regions (Souter et al. 2021; Wicquart et al. 2025), but not in a consistent or permanent manner. This likely reflects the various purposes of monitoring programs (Lindenmayer and Likens 2010; Obura et al. 2019), resulting in regional inferences that are often not based on repeated observations from the same reef locations and likely biased by substantial data gaps.

Automating assessments of reef habitat conditions facilitates the generation of larger and more consistent datasets over time (Obura et al. 2019; Gonzalez‐Rivero et al. 2020; Wicquart et al. 2022; Ditria et al. 2022). Improved use and access to these data, together with new quantitative methods that capture patterns of variability in coral cover, is expected to accelerate our ability to assess changes in coral cover trends (as the key indicator of coral reef habitat condition) with greater robustness. ReefCloud is a digital platform designed to support decision making for the management and conservation of coral reefs using data‐driven products (Australian Institute of Marine Science (AIMS) 2024). The pipeline uses machine learning algorithms to analyze underwater reef images of the seafloor (Gonzalez‐Rivero et al. 2020; Wyatt et al. 2025) and tracks changes in reef habitat conditions with statistical modelling methods. In this paper, we introduce an analytical framework that integrates a state‐of‐the‐art spatio‐temporal model and weighted spatial aggregation into the ReefCloud pipeline to predict coral cover trends at multiple spatial scales and quantify their associations with disturbances while ensuring that uncertainty is appropriately estimated across spatial scales. We demonstrate the capability of the prediction framework using case studies from the central region of the Great Barrier Reef in Australia and American Samoa.

## 1 The Spatio‐Temporal Prediction Framework

### 1.1 Benthic Monitoring Data

Coral cover was estimated from downward‐looking underwater photoquadrats using a convolutional neural network and point‐sampling methodology (Gonzalez‐Rivero et al. 2020; Wyatt et al. 2025). The neural network classified a total of 50 points per image, and these classifications were used to estimate the coverage of benthic communities on the image scale. Underwater images were taken along line transects of varying lengths. We defined observed coral cover at the transect scale as the proportion of points classified as hard coral across all images collected along that transect.

### 1.2 Local Spatial Scale

The finest scale of prediction employed a surface tessellation of 5 km^2^ hexagonal cells as they are known to improve the representation of complex spatial processes like neighbour distances and disturbance impacts (Birch et al. 2007). These gridded locations, hereafter referred to as predictive cells, were delineated using the Tropical Coral Reefs of the World global map (Burke et al. 2011) and the Allen Coral Atlas (Allen Coral Atlas 2022). Predictive cells were constrained to shallow coral habitats (0–12 m), areas where scleractinian hard corals and the majority of coral reef monitoring programs occur, ensuring model predictions align with the spatial distribution of reef‐building corals. The total reef area within each cell was calculated by summing the areas of all reefs contained within that cell. By aggregating monitoring data into these uniform 5 km^2^ cells, we standardized spatial units for modelling purposes and ensured computational feasibility at large spatial scales. However, this aggregation results in a loss of the finer‐scale variability in coral cover and reduces the ability to explicitly represent uncertainty at finer spatial resolutions, such as the transect or reef level.

### 1.3 Disturbances

The framework considered two types of disturbances, tropical cyclones and heat stress exposure. They were selected because of their global data availability and their well‐documented direct and drastic impacts on coral cover across large areas (Harmelin‐Vivien 1994; Hoegh‐Guldberg 1999; Adjeroud et al. 2009; Cheal et al. 2017; Hughes et al. 2018; Puotinen et al. 2020; Eakin et al. 2026). The first metric, cyclone exposure, defined the spatial distribution of likely cyclone impacts estimated using the 4 MW model (Puotinen et al. 2016). Cyclone exposure is defined as the duration of damaging waves (in hours) from cyclones in any given year and was estimated using the duration and speed of modelled cyclonic winds. Potential destructive waves were measured as the average of the highest third of wave heights during a period of strong winds, with wave heights of four meters or more. The longest annual duration of potential damaging wave conditions was treated as the disturbance value for any given year.

The second disturbance metric, Degree Heating Weeks (DHW), was used to quantify exposure to heat stress that is potentially linked to mass coral bleaching events. DHW values were obtained from the Coral Bleaching HotSpot product, which provides near‐real‐time estimates of accumulated thermal stress over a rolling 12‐week period (Liu et al. 2018). For modelling purposes, the annual maximum DHW was used to represent the intensity of a heat stress event for a given year.

Cyclone exposure and DHW values were available at the local scale. When the annual maximum occurred after the field observations in a given year, the observation in that year was assigned to the following year to ensure temporal consistency. Disturbance values were incorporated into the model across multiple time lags (up to 2 years) to account for their potential delayed influence on coral recovery.

### 1.4 Predictive Model

We developed a predictive model to: (1) predict changes in coral cover across space and time at the local scale; (2) quantify uncertainty in a statistically sound manner; (3) estimate disturbance effects; and (4) minimize computing resources. We modelled coral cover data using a Fixed‐Rank Kriging (FRK) approach, which jointly represents space and time (Cressie and Johannesson 2008; Wikle et al. 2019; Zammit‐Mangion and Cressie 2021; Sainsbury‐Dale et al. 2024). FRK accounts for spatio‐temporal dependencies using basis functions and correlated random effects. The spatial basis functions are represented using a low‐rank approximation, characterized by a fixed number of pre‐defined basis functions. Because this number remains constant regardless of the size of the dataset, the method allows for efficient computation with large datasets and spatial domains (Cressie and Johannesson 2008) and therefore circumvents computational challenges previously associated with spatio‐temporal statistics (Wikle 2015).

The spatio‐temporal basis functions are constructed as tensor products of spatial basis functions at multiple spatial scales and temporal basis functions (Zammit‐Mangion and Cressie 2021). Their associated random weights induce spatio‐temporal dependence through a covariance matrix governing the random effects, which in turn defines the dependence between locations. The specification of spatio‐temporal basis functions is automatically generated in the FRK package (Zammit‐Mangion and Cressie 2021).

This modelling approach is ideal for capturing the spatio‐temporal variability in coral cover data. It also naturally handles sampling inconsistencies in monitoring datasets and predicts coral cover at unobserved space–time locations, like unsampled cells and missing years. The model was included within a regression framework to quantify effects of disturbances as fixed effects. Uncertainty associated with predicted coral cover was quantified through Monte Carlo sampling from predictive distributions at local scale. Model parameters were estimated as the posterior mode; these estimates were comparable to maximum likelihood estimates. The predictive model was fitted using the FRK package version 2.3.1 (Sainsbury‐Dale et al. 2024) on the R statistical software version 4.4.1 (R Core Team 2024).

In this study, our response variable is coral count at the local scale, obtained by summing the coral count in all transects that fall into the respective cell. $Z_{i}$ denoted the coral count in a year $t_{i}$ at the cell location $s_{i}$, where i=1,…,n, and n is the number of predictive cells containing coral cover data. The predictive model is given by;

(1) $$Z_{i}∼\text{Binomial}\left(N_{i},Y\left(s_{i},t_{i}\right)\right)$$

(2) $$\text{logit}\left(Y\left(s_{i},t_{i}\right)\right)=x{\left(s_{i},t_{i}\right)}^{⊤}\alpha +v\left(s_{i},t_{i}\right)+\zeta \left(s_{i},t_{i}\right)+R\left(s_{i}\right)$$

Here, $Y\left(s,t\right)$ is a non‐linear function that represents the underlying spatio‐temporal processes influencing the proportion of coral cover at cell i, and $N_{i}$ is the total number of image points summed across all the images in cell i. The $N_{i}$ parameter controls for sample size and reflects the sampling effort associated with transect length. $Y\left(s_{i},t_{i}\right)$ is related to the observed count $Z_{i}$ via a logit transformation.

The model formulation includes between two to four sources of spatio‐temporal variability. The first source of variability is attributed to spatially referenced heat stress and cyclone exposure disturbances at lags 0, 1 and 2 years in the matrix $x\left(s_{i},t_{i}\right)$, with corresponding predictive effects, α vector including the intercept. The inclusion of a disturbance into the model formulation was based on their distributions at multiple spatial scales. Specifically, a disturbance variable was retained when its 75th percentile exceeded zero across the spatial domain of interest and when the absolute pairwise Pearson correlation coefficients among disturbance time lags were below 0.7, indicating an absence of strong collinearity. The second and third sources are a large‐scale variation associated with spatio‐temporal basis functions across the whole domain of prediction, $v\left(s_{i},t_{i}\right)$, and a fine‐scale variation at local scale, $\zeta \left(s_{i},t_{i}\right)$, that are always included in the model. The fourth source of variability is associated with independent and identically distributed reef‐level random effects, $R\left(s_{i}\right)$. Coral reefs are identified using the Tropical Coral Reefs of the World map (Burke et al. 2011) and intersected with each predictive cell to assign reef identifiers. When multiple reefs fall within a single predictive cell, each reef is retained and assigned a unique identifier, which is then used as a categorical random effect.

Model performance was assessed using visual and statistical diagnostics, including model fit and residuals using the DHARMa R package (Hartig 2024). In addition, we developed a “leave‐out data” approach to assess the predictive performance of the model. We used four performance measures to evaluate model predictions with the exclusion of data in two configurations (randomly and by blocks; (Roberts et al. 2017)). This approach enabled us to examine the influence of disturbance presence or absence and the inclusion of random effects on the model predictive performance. It also allowed us to explore the contribution of basis function sizes to model predictions and disturbance size effects.

### 1.5 Weighted Spatial Aggregation

**FIGURE 1:**
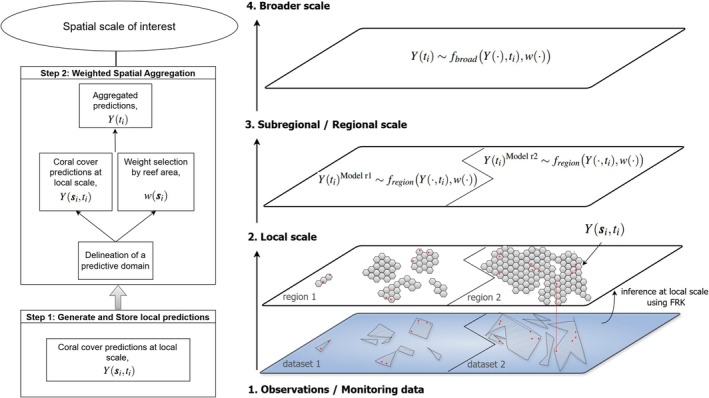
Schematic representation of the spatio‐temporal prediction framework. Coral cover predictions at the local scale (5 km^2^ spatial units) are estimated using the FRK model and stored in the ReefCloud pipeline as predictive distributions. At the scale of a region, predictions are generated through a weighted spatial aggregation approach. The framework is extended to broader spatial scale(s) by combining predictive distributions from multiple regional models.

In our spatio‐temporal prediction framework, coral predictions were aggregated across spatial scales via a systematic integration of model outputs within the ReefCloud pipeline (Figure 1). The first step consisted of generating local predictions of coral cover for all years, $t_{i}$, and predictive cells (5 km^2^ spatial units, $s_{i}$) within a given region, using the predictive model. Local predictions were then stored as posterior predictive distributions, $Y\left(s_{i},t_{i}\right)$, within the ReefCloud pipeline.

For the second step, local predictions were aggregated to estimate trends at broader spatial scales within a hierarchical framework that explicitly propagated uncertainty across space. Before the aggregation, predictive distributions were weighted by the reef area to account for spatial differences in reef extent within each predictive cell and ensure standardized, comparable spatial units. The weighted predictions were subsequently summed across each (sub)region and normalized by the total reef area of that region or subregion to estimate mean coral cover.

For predictions of coral cover trends at other spatial scales, local predictive distributions from multiple (sub)regional models were weighted and aggregated following the same spatial aggregation method described above. Significant year‐to‐year fold changes in coral cover were derived from predictive distributions estimated at multiple spatial scales and represented using arrows. An upward arrow indicates a ≥ 90% probability of increase, a downward arrow a ≥ 90% probability of decrease, and a horizontal arrow no detectable change.

### 1.6 Simulation Experiments

The performance of the model was initially assessed using simulation experiments (Logan and Vercelloni 2025), designed to address two objectives. First, the simulation study allowed us to evaluate the robustness of the predictive model across the entire synthetic spatial domain, rather than at a limited number of randomly chosen locations. This framework enabled the assessment of prediction accuracy at the domain scale by comparing model predictions with the known temporal field used to generate the synthetic data, a quantity that is not available for real‐world datasets. Second, the simulation scenarios evaluated model performance under varying spatial and temporal data resolutions. A common characteristic of coral reef monitoring programs (Gardner et al. 2003; Bruno and Selig 2007; Obura et al. 2019; Souter et al. 2021; Wicquart et al. 2025) is also reflected in the ReefCloud database (Australian Institute of Marine Science (AIMS) 2024), which is dominated by short‐term public datasets (i.e., reef locations surveyed for less than 5 years) and shows geographic biases in monitoring effort (Table S1; Appendix S1). Detailed methods and results of the simulation experiments are presented in Appendix S1.

### 1.7 Association With Disturbances

The association between disturbances and coral cover change was evaluated at the (sub)regional level by examining the α model parameters (effect sizes and corresponding 95% predictive intervals). Disturbance effects were investigated further when the 95% predictive interval of an effect size was entirely below zero, indicating evidence of a negative association with coral cover change. We also combined these model parameters with observed range of disturbance values to predict coral cover across increasing disturbance intensity and to characterize the shape and magnitude of the predicted coral cover responses. A similar spatial approach was used to predict disturbance‐specific patterns in the spatial distribution of coral cover. This was done by fixing other disturbance variables at values close to zero while varying values of the disturbance of interest. Spatial patterns of predicted coral cover associated with each disturbance were evaluated across its observed range, from absence to maximum values, with changes expressed in absolute terms.

## 2 Use Cases

### 2.1 The Central Great Barrier Reef

**FIGURE 2:**
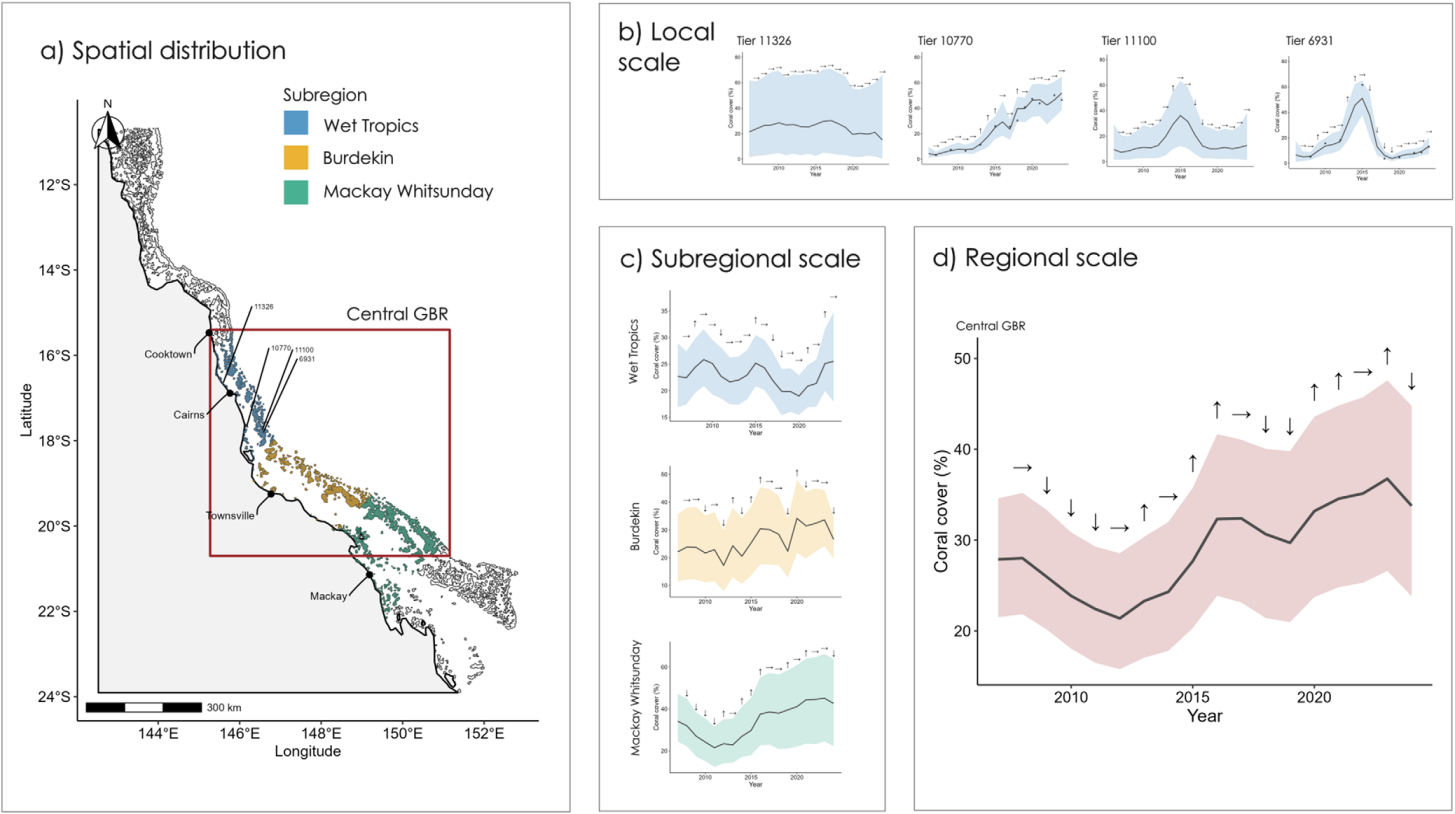
(a) Location of the Central Great Barrier Reef (GBR) region and its three Natural Resource Management (NRM) subregions. Each subregion is divided into predictive cells of 5 km^2^, which define the local scale at which the predictive model estimates coral cover. (b) Four examples of local trends from the Wet Tropics subregion are presented. The black lines show mean predicted coral cover, with shaded areas representing 95% prediction intervals. Dots indicate observed coral cover, and arrows mark significant year‐to‐year changes in coral cover, with at least 90% probability. (c) Three predictive models, one per subregion, are fitted and their predictive distributions of coral cover combined with the weighted spatial aggregation approach to generate subregional trends. (d) Predictions from the three models are weigthed and aggregated using the same approach to estimate the regional trend. Note that the y‐axes are on different scales for visualization purposes. Map lines delineate study areas and do not necessarily depict accepted national boundaries.

We focus first on the central region of the Great Barrier Reef (GBR), which extends from latitude 15.4° to 20.7°S and corresponds to a distinct Marine Ecoregion of the World (MEOW) unit (Spalding et al. 2007). The Central GBR covers a reef area of 5300 km^2^ and comprises three Natural Resource Management (NRM) subregions, including the entirety of the Wet Tropics and Burdekin NRM regions, as well as approximately 60% of the Mackay Whitsunday NRM unit (Figure 2). Image‐based coral cover data were extracted from the ReefCloud database on 01 December 2025, comprising 358 coral reefs and 1728 transects surveyed between 2006 and 2024 at depths of 2–10 m. Reefs in this region benefit from extensive monitoring across multiple programs with varying temporal resolutions, with three of five monitoring programs having survey records spanning more than 15 years (Tables S1–S3; Appendix S2) for each subregion.

The Central GBR was subject to frequent disturbances (Done 1982; Hughes 1992; Osborne et al. 2011; De'Ath et al. 2012; Vercelloni et al. 2017; Mellin et al. 2019; Emslie, Logan, et al. 2024), including multiple heat stress events and cyclones across the three subregions over the past 18 years (Figures S3, S6, and S9 in Appendix S2). The Wet Tropics experienced substantially higher thermal stress, with the maximum value of mean DHWs across data‐cells reaching 12, compared to a maximum of 8 in the other subregions. The maximum mean duration of exposure to cyclonic waves at data‐cells was nearly twice as long in the Mackay Whitsunday region compared to the other regions, associated with the 2017 Cyclone Debbie. In the Wet Tropics, disturbance values at data‐cells reflected the broader regional signal. In the other subregions, however, data‐cells exhibited slightly higher heat stress exposure and lower cyclone exposure relative to the broader regional conditions.

#### 2.1.1 Trends Across Spatial Scales

The predictive model was fitted at the scale of the subregions, rather than the central GBR region, due to its very large spatial extent, resulting in three individual models. Across the subregions, the model formulations that provided the best fit to the observed data accounted for four sources of variability, including heat stress and cyclone exposure at lags of 0, 1, and 2 years. The models exhibited strong predictive performance, with close agreement between predicted and observed coral cover and *R* ^2^ values ranging from 0.79 to 0.89 (Figure S26; Appendix S2). The weighted spatial aggregation method was used to estimate coral cover trends at the subregional and regional scales.

At the local scale, predicted coral cover displayed spatial and temporal variation within each subregion, associated with different levels of uncertainty range (Figure 2; Figures S11–S16 in Appendix S2). The Wet Tropics subregion exhibited a lower overall uncertainty range (defined as the difference between the upper and lower 95% predictive interval bounds) than the Burdekin subregion, which maintains high uncertainty in its southern extent across all years. The Mackay Whitsunday subregion exhibited a temporal increase in uncertainty from 2017 onwards. The propagation of these differences in uncertainty was evident at the subregional level, with the Wet Tropics showing lower uncertainty, as indicated by narrower 95% prediction intervals relative to the other two subregions (Figure 2).

At broader spatial scales, the predictive models captured some fluctuations in coral cover; however, the relatively large uncertainty associated with these estimates limited our ability to detect robust trend changes for most years. Nevertheless, the framework allowed us to detect significant changes associated with decline and recovery in coral cover. At the subregional scale, Wet Tropics coral cover fluctuated between 20% and 25%, with few significant declines in 2011 and 2017–2018 and increases in 2008, 2015, 2021, and 2023 (Figure 2). In the Burdekin, coral cover ranged from 18% to 32%, corresponding to the greatest temporal variability over the past 18 years across the subregions. A significant recovery was estimated between 2015 and 2016, followed by a gradual decline that became significant in 2019, a short recovery in 2020, and a subsequent decline again in 2024. In the Mackay Whitsunday subregion, coral cover declined from 35% to 20% between 2008 and 2011, followed by a period of increase to approximately 40% over the subsequent decade, including six significant increases during this period. At the regional scale, coral cover across the central GBR fluctuated between approximately 20% and 35%. The prediction framework identified two periods of decline (2007–2009 and 2018–2019) and two periods of recovery (2013–2016 and 2020–2023) over the study period.

#### 2.1.2 Association With Disturbances

**FIGURE 3:**
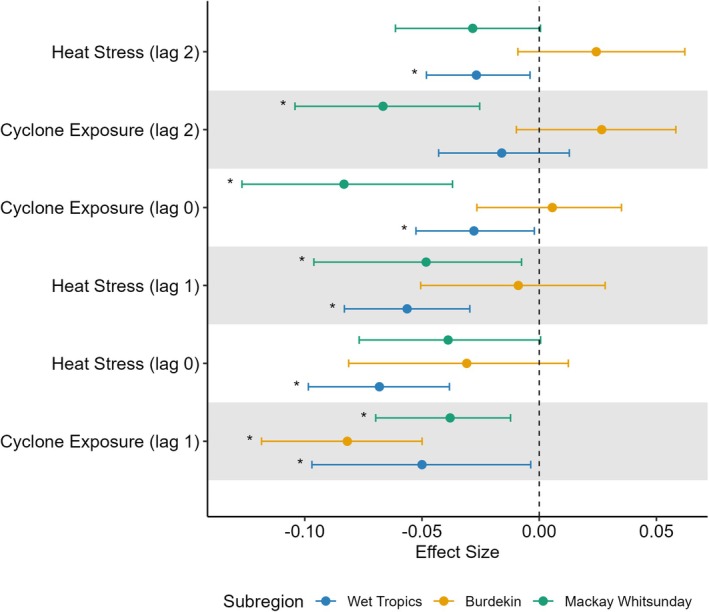
Predicted effect sizes of disturbances. The dots represent the predicted average effect and the intervals represent the corresponding 95% predictive intervals. Stars indicate evidence of a negative effect with predictive intervals below zero.

Model estimates indicated negative associations between disturbances and coral cover change across all subregions, with 95% prediction intervals that did not overlap zero (Figure 3). Five of six disturbance variables were associated with coral cover decline in the Wet Tropics, with heat stress at lags 0 and 1 years showing the strongest mean negative effects among all disturbance–lag combinations, followed by cyclone exposure at lag 1. In the Mackay Whitsunday subregion, four disturbance variables were associated with coral cover change, with the strongest negative mean effects associated with cyclone exposure at lags 0 and 2 years among all disturbance–lag combinations in that subregion. In the Burdekin, coral cover decline was associated with cyclone exposure at lag 1 year.

Increasing heat stress was associated with a linear decline in predicted coral cover across all subregions (Figures S17, S20, and S23 in Appendix S2). Over the range 0–12 DHW, heat stress effects corresponded to declines in predicted coral cover ranging from 11% in the Mackay Whitsunday subregion (Figure S23 in Appendix S2) to 18.3% in the Wet Tropics (Figure S17 in Appendix S2).

Increasing cyclone exposure was associated with a non‐linear decline in predicted coral cover across all subregions, characterized by a steep initial decrease within the first 10–15 h of exposure (Figures S17, S20, and S23 in Appendix S2). Across all subregions and time lags, the magnitude of predicted coral cover decline associated with cyclone exposure was consistently larger than that associated with heat stress when comparing maximum exposure levels within each disturbance type. Cyclone‐associated declines ranged from approximately 38% to 44%, evaluated at maximum observed exposure durations of 30–45 h.

Spatial patterns of absolute changes associated with individual disturbances show relatively smaller predicted declines in coral cover at the local scale across all subregions, with some variation among subregions and disturbance types (Figures S18, S19, S21, S22, S24, and S25 in Appendix S2). This pattern refers specifically to model estimates in which each disturbance is evaluated independently while holding other disturbances constant. In contrast, models in which multiple disturbances are allowed to vary jointly produce larger estimated declines in coral cover (Figures S17, S20, and S23 in Appendix S2).

### 2.2 American Samoa

**FIGURE 4:**
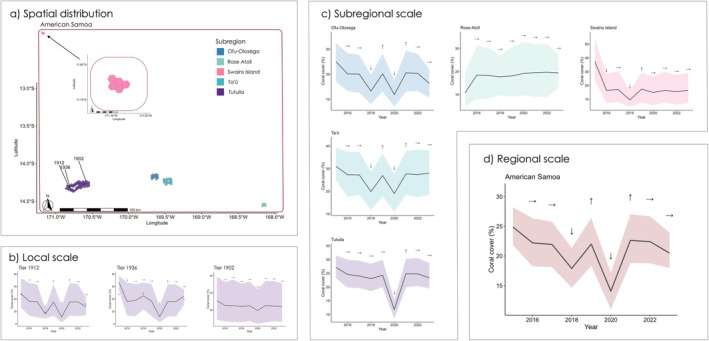
(a) Location of the American Samoa region and its islands and atolls representing the subregions. The region is divided into predictive cells of 5 km^2^, which define the local scale at which the predictive model estimates coral cover. (b) Four examples of local trends from Tutuila subregion are shown. The black lines show mean predicted coral cover, with shaded areas representing 95% prediction intervals. Dots indicate observed coral cover, and arrows mark significant year‐to‐year changes in coral cover, with at least 90% probability. (c) Predictive distributions of coral cover are combined with a weighted spatial aggregation approach to generate subregional trends. (d) Predictions from the entire spatial domain are weigthed and aggregated using the same approach to estimate the regional trend. Note that the y‐axes are on different scales for visualization purposes. Map lines delineate study areas and do not necessarily depict accepted national boundaries.

The second use case focuses on American Samoa, located in Polynesia in the South Central Pacific Ocean. The country has a reef area of 59.2 km^2^ and is composed of a unique MEOW region with three islands and two atolls, considered as subregions in this study (Figure 4). Image‐based coral cover data were extracted from the ReefCloud database on 15 March 2026 including a total of 507 transects deployed across all islands and atolls and surveyed in 2015, 2018 and 2023, at depths of 3–10 m (Table S1; Appendix S3). These data come from a single monitoring program led by the National Oceanic and Atmospheric Administration (NOAA) as part of the U.S. Pacific Islands National Coral Reef Monitoring Program (National Oceanic and Atmospheric Administration (NOAA) 2025).

American Samoa was exposed to five heat stress events and two cyclones between 2015 and 2023 (Figure S4; Appendix S3). While environmental data were available for all disturbance events, few of these events were captured by the benthic monitoring program. Monitoring observations were available only after the 2015 heat stress event, which had a mean DHW of 8, consistent with the regional signal. Similarly, benthic monitoring data were available for only one cyclone event, for which slightly higher mean exposure to cyclonic waves was recorded at data cells compared to the corresponding regional mean conditions over the same period (Figure S4; Appendix S3).

#### 2.2.1 Trends Across Spatial Scales

We fitted the predictive model to the American Samoa region using four sources of variability, including heat stress and cyclone exposure at lag 0 only, as benthic observations were not available at 1 and 2 years time lags. The predictive performance of the model was lower than for the central Great Barrier Reef subregions with a *R* ^2^ of 0.55 (Figure S15; Appendix S3). The weighted spatial aggregation method was used to estimate coral cover trends at the subregional and regional scales.

**FIGURE 5:**
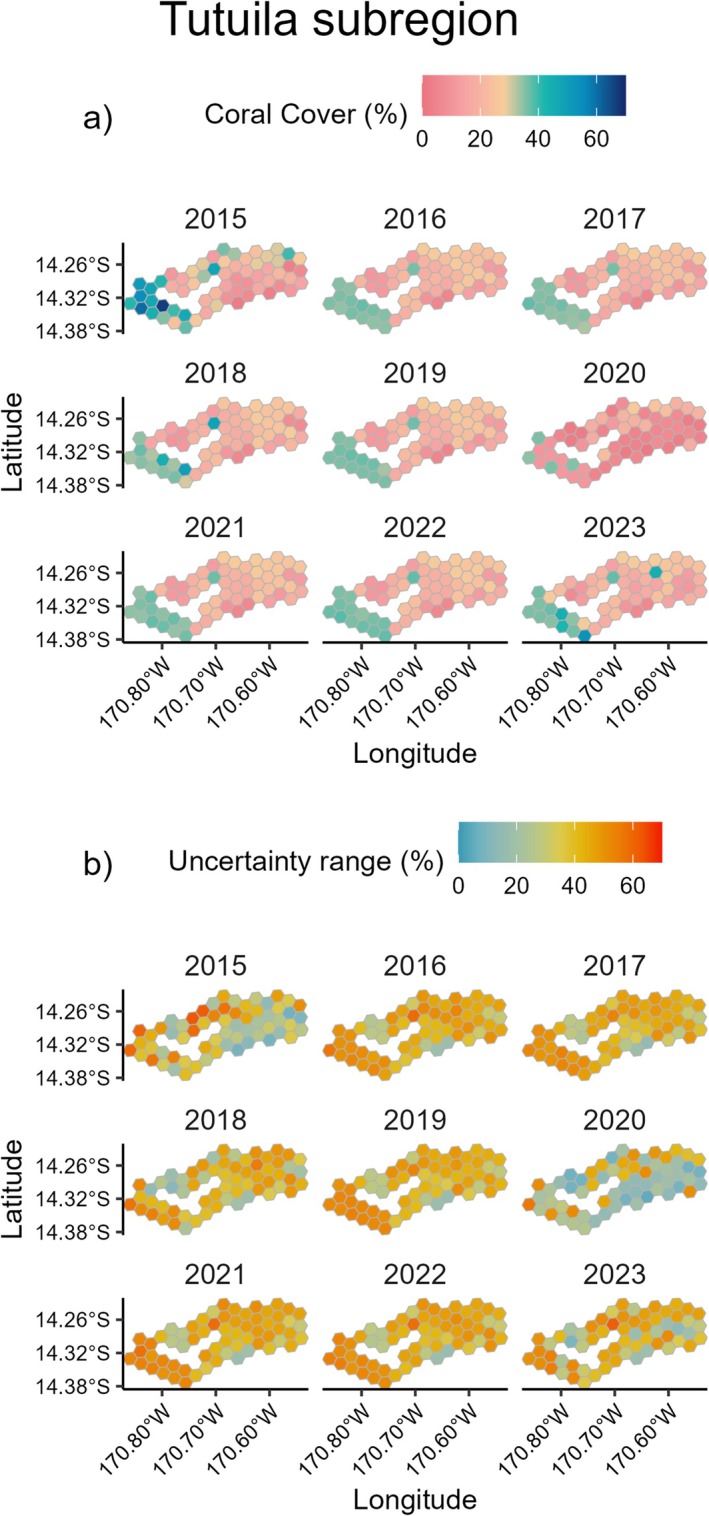
(a) Predicted coral cover across Tutuila subregion with values correspond to the posterior means between 2015 and 2023 and (b) associated uncertainty range for each year. Values correspond to the range between the lower and upper bounds of the 95% posterior predictive intervals of the estimated coral cover.

At the local scale, predicted coral cover was approximately 20% across the five subregions with varying temporal changes (Figure 4), with values around 40% observed in the southwest of the Tutuila subregion in most years (Figure 5) and in Swains Island only for 2015 (Figure S10; Appendix S3). Uncertainty ranges showed low spatial and temporal variation (Figure 5), remaining relatively homogeneous across subregions and years (Figures S6–S10 in Appendix S3).

At the subregion scale, coral cover exhibited broadly similar trends across the three islands, with a significant decline estimated in Tutuila in 2020, whereas Ofu–Olosega and Ta'ū showed smaller but significant declines as well in 2018 and 2020 (Figure 4). On Swains Island, predicted coral cover declined significantly in 2016 and 2018, from approximately 40% to 18%, followed by recovery in 2019. In contrast, no discernible trend was captured by the model for Rose Atoll for the period 2015–2023. At the regional scale, predicted coral cover varied between 25% and 15%, with two significant declines estimated in 2018 and 2020, the latter being more pronounced. In both cases, these declines were followed by increases in the subsequent year (Figure 4).

#### 2.2.2 Association With Disturbances

Despite a weak disturbance signal in the data (Figure S4; Appendix S3), the model estimated a negative effect of cyclone exposure on coral cover change (Figure S11; Appendix S3). Increasing cyclone exposure duration was associated with a linear decline in coral cover over durations of 0–15 h, decreasing from approximately 50% to 35%, with relatively large uncertainty (Figure S12; Appendix S3). Spatial patterns of absolute change associated with maximum cyclone exposure across American Samoa indicated declines ranging from 5% to 27%, with variation among subregions. The southern part of Tutuila exhibited the largest predicted declines in coral cover relative to the other islands and atolls (Figures S13 and S14 in Appendix S3).

## 3 Discussion

In this study, we present a comprehensive prediction framework to estimate coral cover trends across multiple spatial scales, ranging from a local scale of 5 km^2^ to a broad region exceeding 5000 km^2^. Through the use cases, we demonstrate that the prediction framework can generate new insights from monitoring data across varying spatial and temporal data resolutions, by estimating coral cover trends at scales relevant to regional decision making, attributing changes to environmental disturbances and quantifying the influence of uncertainty on the robustness of trend detection.

### 3.1 The ReefCloud Platform

Despite ongoing efforts and international calls to expand reef monitoring worldwide, locations with widespread and regular coral reef monitoring remain concentrated in a relatively small number of regions (Souter et al. 2021). The ReefCloud platform has been developed to address this issue by enabling public access to three components: data coverage, analysis, and reporting. First, its machine learning algorithms for management and processing of underwater reef images allow users to generate ecological data in a cost‐ and time‐efficient manner, thereby easing monitoring efforts and increasing data availability (Gonzalez‐Rivero et al. 2020; Australian Institute of Marine Science (AIMS) 2024; Wyatt et al. 2025). Second, these data feed a statistical model that predicts annual hard coral cover at the site level. Third, data summaries and model outputs are presented on a public dashboard, allowing users to assess coral reef habitat condition and promote collaboration by enabling regional comparisons and sharing insights across institutions and sectors. In the ReefCloud platform, data are considered public when stakeholders have consented to display them on the online dashboard and to include them in analytical processes. The prediction framework proposed here complements the second component by providing additional information on coral cover trends across multiple spatial scales, uncertainty, and disturbance effects. Monitoring observations at the transect scale are aggregated within predictive cells, ensuring that the raw data remain confidential. Making the model outputs accessible to all users supports evidence‐based management and may encourage more contributors to make additional datasets publicly available. Over time, these contributions will enhance the value and robustness of the ReefCloud components.

### 3.2 The Spatio‐Temporal Prediction Framework

Insights gained from decades of data highlight the importance of long‐term coral reef monitoring for detecting robust trends and attributing the key drivers of coral cover change (Fine et al. 2019; Obura et al. 2019; Donovan et al. 2021; Emslie, Logan, et al. 2024; Edmunds 2024; Wicquart et al. 2025). However, studies of coral reef habitat condition rarely account for spatial dependence in long‐term data analyses; but see (Edmunds and Bruno 1996; Peterson et al. 2020; Roelfsema et al. 2021; Edmunds and Smith 2022; Vercelloni et al. 2023; Srednick et al. 2023), which may lead to the underestimation of uncertainty in predicted trends (Johnson et al. 2024) and likely bias inference on disturbance effects.

In the framework presented here, spatio‐temporal dependencies are represented by a latent process that is not directly observed but inferred from the data, in contrast to the explanatory variables which are directly measured. By incorporating spatial and temporal correlation structures, the model captures dependencies among observations, reflecting that nearby reef locations respond similarly and that this similarity evolves over time (Wikle et al. 2019). These dependencies can support more robust predictions at unmonitored locations within the study domain and enhance the quantification of associated uncertainty (Cressie and Johannesson 2008; Zammit‐Mangion and Cressie 2021; Sainsbury‐Dale et al. 2024; Johnson et al. 2024). By tracking coral cover continuously across space and time (within the range of the observed data), quantifying uncertainty directly from the data and accounting for disturbances, more complete and representative patterns of change can be captured which can improve the robustness of trend change detection and reduce the risk of false positives (when a trend change is detected but with high uncertainty).

The integration of the statistical model into the ReefCloud pipeline is tailored to the need of tracking coral changes across multiple spatial scales. By integrating predictive distributions at local scale into the pipeline, we demonstrate the scalability of the approach, enabling predictions at relevant scales, from broad regions such as the central GBR in Australia to finer scales such as the American Samoa islands and atolls. The weighted aggregation approach ensures the standardization of coral cover predictions while explicitly accounting for the spatial heterogeneity of coral reefs which, in our case, is also key to propagating uncertainty across spatial scales in a statistically robust manner. A similar weighting approach is already used by the Global Coral Reef Monitoring Network (GCRMN) in its synthesis of global and regional changes in coral cover (Souter et al. 2021; Wicquart et al. 2025). With the spatio‐temporal prediction framework fully developed and tested, the next step is to automate the approach for application across many regions.

### 3.3 Limitations

The application of the framework to the two use cases highlights several limitations that should be considered for broader applications. The prevalence of short‐term datasets in the ReefCloud database can reduce the predictive performance of the model, potentially underestimating local scale uncertainty and limiting the ability to draw robust inferences about the effects of disturbances (Appendix S1; Figures S11 and S12 in Appendix S3). In contrast, geographical biases, where long‐term monitoring is concentrated in small areas within a vast region, contribute to increased local uncertainty at locations far from the observed data, even when disturbances are relatively well represented in the data like in the central GBR (Figure 2; Figures S11–S16 in Appendix S2). We also show that local uncertainty can be substantial even at locations relatively close to existing data when observations are fragmented, as exemplified by the American Samoa use case (Figure 4). Despite high spatial sampling density within each subregion (Figure S2; Appendix S3), the opportunistic sampling across years limited the ability of the model to capture disturbance effects, leading to increased uncertainty. For example, the model attributed coral cover declines to cyclone exposure (Figure S11; Appendix S3) and predicted a large regional decline in 2020, when 75% of the region was exposed to cyclonic waves (Figure S4; Appendix S3). However, the absence of coral cover observations in 2020 meant that the relationship between cyclone exposure and coral cover change was learned primarily from 2018 data. Cyclone exposure in 2020 was approximately twice as long as in previous events, so the model was required to extrapolate beyond the observed range. As a result, these predictions and cyclone effect size should be interpreted with caution. Additional criteria will need to be incorporated into model variable selection, not only with respect to their regional distribution and potential collinearity, but also to ensure that monitoring data adequately capture the range of disturbance conditions over the spatio‐temporal prediction domains, particularly in regions with limited short‐term monitoring programs.

The prediction framework can only incorporate disturbances that are continuously available at the local scale, both to predict unsampled cells and to enable automation across many regions. Consequently, the inclusion of additional acute disturbances, such as crown‐of‐thorns starfish outbreaks, other coral predators, and coral disease outbreaks, as well as chronic stressors, including long‐term changes in water quality, and localized human impacts, remains challenging, even though they could improve predictions and strengthen attribution of coral cover changes (Connell et al. 1997; Bruno and Selig 2007; Osborne et al. 2011; De'Ath et al. 2012; Kayal et al. 2012; Mellin et al. 2019; Peterson et al. 2020; Castro‐Sanguino et al. 2021; Walker et al. 2024; Emslie, Logan, et al. 2024; McClanahan et al. 2024). The incorporation of ambient environmental conditions including sea surface temperature and climatological metrics may also improve predictions, particularly at unsampled cells. As shown in Figures 2 and 4, unsampled cells are characterized by flat trends concentrated toward the (sub)regional mean coral cover and are associated with large uncertainty. This suggests that heat stress and cyclone exposure, including their associated lagged effects, have limited predictive power in these predictive locations, likely because they provide little information during non‐disturbance years. This limitation restricts the conclusions that can be drawn from the framework, as it focuses only on two acute disturbances that have a drastic impact on coral cover across large reef areas. Additional research is needed to incorporate ambient environmental conditions into the model while balancing improvements in predictive performance with model interpretability.

### 3.4 Future Perspectives

By estimating trends and disturbance effects, the model can guide users in selecting additional monitoring locations where logistically possible, either in space (e.g., new sites) or in time (e.g., repeated observations) and improve the spatial coverage of observations and their alignment with disturbance events. In particular, areas characterized by high predictive uncertainty may be prioritized for additional sampling to reduce uncertainty in model estimates. In addition, when a significant change is detected at the (sub)regional scale (indicated by a downward or upward arrow, Figures 2 and 4), this reflects a period of instability associated with either decline or recovery in coral cover. These signals can be used to guide targeted resampling of the same locations to verify whether subsequent observations confirm, attenuate, or reverse the detected change (Castro‐Sanguino et al. 2021). During periods of stability and/or lack of detectable changes from predicted coral trends (indicated by horizontal arrow), monitoring efforts could also be shifted to additional sites associated with high predictive uncertainty. The selection of new monitoring sites is guided by multiple factors, including their distance from existing monitoring locations and the disturbance values recorded in previous years. It is important to consider the spatio‐temporal trade‐offs involved, particularly when repeated observations are essential for producing robust long‐term trends (Hughes 1992; Lindenmayer et al. 2022; Edmunds 2024; Emslie, Ceccarelli, et al. 2024). The decision to establish additional sites or to prioritize repeated observations will likely need to be made on a case‐by‐case basis by the local reef scientists, managers and stakeholders.

Finally, an important avenue for future development is the extension of the framework to additional benthic groups including macroalgae and soft corals which would provide a more comprehensive understanding of shallow reef dynamics. Beyond coral reefs, the framework could also be adapted for other marine ecosystems where spatio‐temporal standardized monitoring data are available. These extensions would not only broaden the scope of the prediction framework but also enhance its utility for ecosystem‐based management and conservation across local to broad spatial scales.

## Author Contributions

**Julie Vercelloni:** conceptualization, investigation, writing – original draft, methodology, validation, visualization, writing – review and editing, software, data curation, project administration, formal analysis. **Andrew Zammit‐Mangion:** software, formal analysis, methodology, validation, visualization, writing – review and editing, supervision. **Manuel González‐Rivero:** resources, conceptualization, investigation, funding acquisition, writing – review and editing, visualization, project administration, supervision. **Britta Schaffelke:** conceptualization, writing – review and editing, supervision, visualization. **Murray Logan:** methodology, validation, software, formal analysis, supervision, data curation, investigation, conceptualization. **Matthew Sainsbury‐Dale:** software, formal analysis, writing – review and editing, validation, methodology. **Kerrie Mengersen:** conceptualization, investigation, funding acquisition, writing – review and editing, methodology, validation, visualization, software, formal analysis, supervision.

## Conflicts of Interest

The authors declare no conflicts of interest.

## Supporting information


**Appendix S1:** Use case: simulation experiments.
**Table S1:** Summary of public datasets available in the ReefCloud pipeline as of April 2026. Percentages are based on the absolute counts provided in parentheses. Monitoring programs are classified by temporal resolution with short‐term (0–5 years), medium‐term (6–15 years), and long‐term (> 15 years). Data are considered public when stakeholders have consented to include them in analytical analyses. Regions were extracted using the geospatial data of (British Oceanographic Data Centre (BODC) 2023).
**Table S2:** Details of the simulation scenarios.
**Table S3:** Details of predictive measures used to evaluate the predictive performances of the models in the simulation experiments. The metrics yield scores between 0 and 1, where values closer to 0 indicate a better predictive performance.
**Figure S1:** Performance metrics for the four simulation scenarios.
**Figure S2:** Spatial distribution of the synthetic reefs. The red dots indicate monitoring locations and hexagons represent cell boundaries. A cell is considered a data‐cell if it contains a red dot; otherwise, it is classified as a predictive‐cell for the scenario Short‐term.
**Figure S3:** Spatial distribution of the synthetic reefs. The red dots indicate monitoring locations and hexagons represent cell boundaries. A cell is considered a data‐cell if it contains a red dot; otherwise, it is classified as a predictive‐cell for the scenario Medium‐term.
**Figure S4:** Spatial distribution of the synthetic reefs. The red dots indicate monitoring locations and hexagons represent cell boundaries. A cell is considered a data‐cell if it contains a red dot; otherwise, it is classified as a predictive‐cell for the scenario Long‐term.
**Figure S5:** Spatial distribution of the synthetic reefs. The red dots indicate monitoring locations and hexagons represent cell boundaries. A cell is considered a data‐cell if it contains a red dot; otherwise, it is classified as a predictive‐cell for the scenario Temporally‐sparse.
**Figure S6:** Simulated transect‐scale coral cover values (%) within each reef, with colours representing the two sites for the scenario Short‐term.
**Figure S7:** Simulated transect‐scale coral cover values (%) within each reef, with colours representing the two sites for the scenario Medium‐term.
**Figure S8:** Simulated transect‐scale coral cover values (%) within each reef, with colours representing the two sites for the scenario Long‐term.
**Figure S9:** Simulated transect‐scale coral cover values (%) within each reef, with colours representing the two sites for the scenario Temporally‐sparse.
**Figure S10:** Temporal patterns of mean coral cover (%) by site within reef. White gaps show missing data for the scenario Short‐term.
**Figure S11:** Temporal patterns of mean coral cover (%) by site within reef. White gaps show missing data for the scenario Medium‐term.
**Figure S12:** Temporal patterns of mean coral cover (%) by site within reef. White gaps show missing data for the scenario Long‐term.
**Figure S13:** Temporal patterns of mean coral cover (%) by site within reef. White gaps show missing data for the scenario Temporally‐sparse.
**Figure S14:** Regional trend. The black line shows mean values and green shadows 95% predictive intervals. The dotted blue line is the true simulated temporal field estimated from the Gaussian Random Markov Field values in the simulation Short‐term.
**Figure S15:** Regional trend. The black line shows mean values and green shadows 95% predictive intervals. The dotted blue line is the true simulated temporal field estimated from the Gaussian Random Markov Field values in the simulation Medium‐term.
**Figure S16:** Regional trend. The black line shows mean values and green shadows 95% predictive intervals. The dotted blue line is the true simulated temporal field estimated from the Gaussian Random Markov Field values in the simulation Long‐term.
**Figure S17:** Regional trend. The black line shows mean values and green shadows 95% predictive intervals. The dotted blue line is the true simulated temporal field estimated from the Gaussian Random Markov Field values in the simulation Temporally‐sparse.
**Figure S18:** Predictions of coral cover (%) in Short‐term.
**Figure S19:** Predictions of coral cover (%) in Medium‐term.
**Figure S20:** Predictions of coral cover (%) in Long‐term.
**Figure S21:** Predictions of coral cover (%) in Temporally‐sparse.
**Figure S22:** Uncertainty associated with predicted coral cover values in Short‐term.
**Figure S23:** Uncertainty associated with predicted coral cover values in Medium‐term.
**Figure S24:** Uncertainty associated with predicted coral cover values in Long‐term.
**Figure S25:** Uncertainty associated with predicted coral cover values in Temporally‐sparse.
**Figure S26:** Examples of estimated coral trends at the data‐cells. Black lines represent mean predicted coral cover, with shaded areas indicating 95% predictive intervals. Dots show observed coral cover averaged at the cell level, which were used as the response variable in the spatio‐temporal model. For the scenario Short‐term.
**Figure S27:** Examples of estimated coral trends at the data‐cells. Black lines represent mean predicted coral cover, with shaded areas indicating 95% predictive intervals. Dots show observed coral cover averaged at the cell level, which were used as the response variable in the spatio‐temporal model. For the scenario Medium‐term.
**Figure S28:** Examples of estimated coral trends at the data‐cells. Black lines represent mean predicted coral cover, with shaded areas indicating 95% predictive intervals. Dots show observed coral cover averaged at the cell level, which were used as the response variable in the spatio‐temporal model. For the scenario Long‐term.
**Figure S29:** Examples of estimated coral trends at the data‐cells. Black lines represent mean predicted coral cover, with shaded areas indicating 95% predictive intervals. Dots show observed coral cover averaged at the cell level, which were used as the response variable in the spatio‐temporal model. For the scenario Temporally‐sparse.
**Figure S30:** Examples of estimated coral trends at predictive‐cells. Black lines represent mean predicted coral cover, with shaded areas indicating 95% predictive intervals, for the scenario Short‐term.
**Figure S31:** Examples of estimated coral trends at predictive‐cells. Black lines represent mean predicted coral cover, with shaded areas indicating 95% predictive intervals, for the scenario Medium‐term.
**Figure S32:** Examples of estimated coral trends at predictive‐cells. Black lines represent mean predicted coral cover, with shaded areas indicating 95% predictive intervals, for the scenario Long‐term.
**Figure S33:** Examples of estimated coral trends at predictive‐cells. Black lines represent mean predicted coral cover, with shaded areas indicating 95% predictive intervals, for the scenario Temporally‐sparse.
**Figure S34:** Estimated effect sizes of disturbance exposure (in the logit scale). The points represent the estimated effect and the intervals represent the corresponding 95% predictive intervals, for the scenario Short‐term.
**Figure S35:** Estimated effect sizes of disturbance exposure (in the logit scale). The points represent the estimated effect and the intervals represent the corresponding 95% predictive intervals, for the scenario Medium‐term.
**Figure S36:** Estimated effect sizes of disturbance exposure (in the logit scale). The points represent the estimated effect and the intervals represent the corresponding 95% predictive intervals, for the scenario Long‐term.
**Figure S37:** Estimated effect sizes of disturbance exposure (in the logit scale). The points represent the estimated effect and the intervals represent the corresponding 95% predictive intervals, for the scenario Temporally‐sparse.
**Figure S38:** Model goodness‐of‐fit showing predicted coral cover versus true values for the prediction‐cells. True values corresponds to the mean coral cover at cell‐level.
**Figure S39:** Weighted effects of cyclone exposure per surveyed year for the scenario Short‐term.
**Figure S40:** Weighted effects of cyclone exposure per surveyed year for the scenario Medium‐term.
**Figure S41:** Weighted effects of cyclone exposure per surveyed year for the scenario Long‐term.
**Figure S42:** Weighted effects of cyclone exposure per surveyed year for the scenario Temporally‐sparse.
**Figure S43:** Weighted effects of heat stress event per surveyed year for the scenario Short‐term.
**Figure S44:** Weighted effects of heat stress event per surveyed year for the scenario Medium‐term.
**Figure S45:** Weighted effects of heat stress event per surveyed year for the scenario Long‐term.
**Figure S46:** Weighted effects of heat stress event per surveyed year for the scenario Temporally‐sparse.

**Appendix S2:** Use case: central great barrier reef.
**Figure S1:** Locations of the three subregions along the Great Barrier Reef. Red dots indicates the predictive cells containing coral cover observations between 2006 and 2024.
**Table S1:** Summary of public Wet Tropics datasets available in the ReefCloud pipeline as of December 2025. Percentages are based on the absolute counts provided in parentheses. Monitoring programs are classified by temporal resolution with short‐term (0–5 years), medium‐term (6–15 years), and long‐term (> 15 years). Data are considered public when stakeholders have consented to include them in analytical analyses.
**Figure S2:** Temporal pattern of mean coral cover (%) by data‐cells in the Wet Tropics (2006–2024). White gaps represent missing data.
**Figure S3:** Proportion of cells affected by disturbances for all‐cells and data‐cells for each year between 2006 and 2024 (in the case of data‐cells, the proportion is calculated with respect to the number of all‐cells). Colours indicate (a) average Degree Heating Weeks (DHW) and (b) average exposure to cyclone waves.
**Figure S4:** Correlation matrix of cyclone‐exposure and heat‐stress predictors at lags 0, 1, and 2 years. Numbers indicate Pearson correlation coefficients calculated using pairwise complete observations, and variables are ordered using hierarchical clustering with highly correlated variables grouped together. The figure was used to identify highly collinear predictors (|r| > 0.7) and inform model variable selection.
**Table S2:** Summary of public Burdekin datasets available in the ReefCloud pipeline as of December 2025. Percentages are based on the absolute counts provided in parentheses. Monitoring programs are classified by temporal resolution with short‐term (0–5 years), medium‐term (6–15 years), and long‐term (> 15 years). Data are considered public when stakeholders have consented to include them in analytical analyses.
**Figure S5:** Temporal pattern of mean coral cover (%) by data‐cells in Burdekin (2007–2024). White gaps represent missing data.
**Figure S6:** Proportion of cells affected by disturbances for all‐cells and data‐cells for each year between 2007 and 2024 (in the case of data‐cells, the proportion is calculated with respect to the number of all‐cells). Colours indicate (a) average Degree Heating Weeks (DHW) and (b) average exposure to cyclone waves.
**Figure S7:** Correlation matrix of cyclone‐exposure and heat‐stress predictors at lags 0, 1, and 2 years. Numbers indicate Pearson correlation coefficients calculated using pairwise complete observations, and variables are ordered using hierarchical clustering with highly correlated variables grouped together. The figure was used to identify highly collinear predictors (|r| > 0.7) and inform model variable selection.
**Table S3:** Summary of public Mackay Whitsunday datasets available in the ReefCloud pipeline as of December 2025. Percentages are based on the absolute counts provided in parentheses. Monitoring programs are classified by temporal resolution with short‐term (0–5 years), medium‐term (6–15 years), and long‐term (> 15 years). Data are considered public when stakeholders have consented to include them in analytical analyses.
**Figure S8:** Temporal pattern of mean coral cover (%) by data‐cells in Mackay Whitsunday (2007–2024). White gaps represent missing data.
**Figure S9:** Proportion of cells affected by disturbances for all‐cells and data‐cells for each year between 2007 and 2024 (in the case of data‐cells, the proportion is calculated with respect to the number of all‐cells). Colours indicate (a) average Degree Heating Weeks (DHW) and (b) average exposure to cyclone waves.
**Figure S10:** Correlation matrix of cyclone‐exposure and heat‐stress predictors at lags 0, 1, and 2 years. Numbers indicate Pearson correlation coefficients calculated using pairwise complete observations, and variables are ordered using hierarchical clustering with highly correlated variables grouped together. The figure was used to identify highly collinear predictors (|r| > 0.7) and inform model variable selection.
**Figure S11:** Predicted coral cover values across the Wet Tropics for each year between 2006 and 2024. Values correspond to the posterior means.
**Figure S12:** Uncertainty associated with predictions across the Wet Tropics for each year between 2006 and 2024. Values correspond to the range between the lower and upper bounds of the 95% posterior predictive intervals of the estimated coral cover.
**Figure S13:** Predicted coral cover values across Burdekin for each year between 2007 and 2024. Values correspond to the posterior means.
**Figure S14:** Uncertainty associated with predictions across Burdekin for each year between 2007 and 2024. Values correspond to the range between the lower and upper bounds of the 95% posterior predictive intervals of the estimated coral cover.
**Figure S15:** Predicted coral cover values across Mackay Whitsunday for each year between 2007 and 2024. Values correspond to the posterior means.
**Figure S16:** Uncertainty associated with predictions across Mackay Whitsunday for each year between 2007 and 2024. Values correspond to the range between the lower and upper bounds of the 95% posterior predictive intervals of the estimated coral cover.
**Figure S17:** Association with Disturbances. The panels display the predicted coral cover across the range of disturbance values, from the minimum to the maximum values observed in Wet Tropics. Note that values of Heat Stress are expressed in Degree Heating Weeks and Cyclone Exposure in hours. The lines correspond to the averaged predicted values and shaded areas their associated 95% prediction intervals. Effects have been calculated at the mean values of all the other covariates.
**Figure S18:** Spatial predictions of coral cover under fixed low and high levels of heat stress across the Wet Tropics.
**Figure S19:** Associated absolute changes of coral cover between low and high levels of heat stress across the Wet Tropics.
**Figure S20:** Association with Disturbances. The panels display the predicted coral cover across the range of disturbance values, from the minimum to the maximum values observed in Burdekin. Note that values of Heat Stress are expressed in Degree Heating Weeks and Cyclone Exposure in hours. The lines correspond to the averaged predicted values and shaded areas their associated 95% prediction intervals. Effects have been calculated at the mean values of all the other covariates.
**Figure S21:** Spatial predictions of coral cover under fixed low and high levels of cyclone exposure at lag 1 year across Burdekin.
**Figure S22:** Associated absolute changes of coral cover between low and high levels of cyclone exposure at lag 1 year across Burdekin.
**Figure S23:** Association with Disturbances. The panels display the predicted coral cover across the range of disturbance values, from the minimum to the maximum values observed in Mackay Whitsunday. Note that values of Heat Stress are expressed in Degree Heating Weeks and Cyclone Exposure in hours. The lines correspond to the averaged predicted values and shaded areas their associated 95% prediction intervals. Effects have been calculated at the mean values of all the other covariates.
**Figure S24:** Spatial predictions of coral cover under fixed low and high levels of cyclone exposure across Mackay Whitsunday.
**Figure S25:** Associated absolute changes of coral cover between low and high levels of cyclone exposure across Mackay Whitsunday.
**Figure S26:** Model goodness‐of‐fit for the three subregions. Coral cover observations at the local scale were used to fit the spatio‐temporal models. The R2 statistic measures how well the predicted values reproduce the observations, with values closer to 1 indicating a better fit along the 1:1 red dotted line.
**Figure S27:** QQ plot residuals, dispersion and deviation diagnostics.
**Figure S28:** Metrics of model predictive performance by tests. Each axis represents one of the evaluation metrics described above. The length of each arm represents the model predictive performance on that metric for a given test, with values scaled from 1 (poor) to 0 (best). Overall, better predictive performances of the models are indicated by smaller polygons.
**Figure S29:** Locations of the basis functions for each model with (a) Default, (b) 5, (c) 3 and (d) 1.
**Figure S30:** Regional trends predicted by the spatio‐temporal models. Solid lines represent mean predicted coral cover, with shaded areas indicating predictive intervals.
**Figure S31:** Estimated effect sizes of disturbance effects. The points represent the estimated effect and the intervals represent the corresponding 95% confidence intervals.

**Appendix S3:** Use case: american samoa.
**Figure S1:** Locations of the five islands across the American Samoa. Red dots indicates the data cells containing coral cover observations between 2015 and 2023.
**Figure S2:** Zoom‐in on the subregions. Red dots indicates the data cells containing coral cover observations between 2015 and 2023.
**Table S1:** Summary of public American Samoa datasets available in the ReefCloud pipeline as of March 2026. Percentages are based on the absolute counts provided in parentheses. Monitoring programs are classified by temporal resolution with short‐term (0–5 years), medium‐term (6–15 years), and long‐term (> 15 years). Data are considered public when stakeholders have consented to include them in analytical analyses.
**Figure S3:** Temporal pattern of mean coral cover (%) by data cells in the American Samoa (2015–2023). White gaps represent missing data.
**Figure S4:** Proportion of regional areas affected by disturbances across two data levels all‐cells and data‐cells for each year between 2015 and 2023 (in the case of data‐cells, the proportion is calculated with respect to the number of all‐cells). Colours indicate (a) average Degree Heating Weeks (DHW) and (b) average exposure to cyclone waves.
**Figure S5:** Correlation matrix of cyclone‐exposure and heat‐stress predictors at lag 0 years. Numbers indicate Pearson correlation coefficients calculated using pairwise complete observations, and variables are ordered using hierarchical clustering with highly correlated variables grouped together. The figure was used to identify highly collinear predictors (|r| > 0.7) and inform model variable selection.
**Figure S6:** (a) Predicted coral cover values across Tutuila subregion and (b) associated uncertainty range for each year between 2015 and 2023. Values correspond to their psoterior means and the lower and upper bounds of the 95% posterior credible intervals, respectively.
**Figure S7:** (a) Predicted coral cover values across Ofu‐Olosega subregion and (b) associated uncertainty range for each year between 2015 and 2023. Values correspond to their psoterior means and the lower and upper bounds of the 95% posterior credible intervals, respectively.
**Figure S8:** (a) Predicted coral cover values across Ta'ū subregion and (b) associated uncertainty range for each year between 2015 and 2023. Values correspond to their psoterior means and the lower and upper bounds of the 95% posterior credible intervals, respectively.
**Figure S9:** (a) Predicted coral cover values across Rose Atoll subregion and (b) associated uncertainty range for each year between 2015 and 2023. Values correspond to their psoterior means and the lower and upper bounds of the 95% posterior credible intervals, respectively.
**Figure S10:** (a) Predicted coral cover values across Swains Island subregion and (b) associated uncertainty range for each year between 2015 and 2023. Values correspond to their psoterior means and the lower and upper bounds of the 95% posterior credible intervals, respectively.
**Figure S11:** Predicted effect sizes of disturbances. The dots represent the predicted averaged effect and the intervals represent the corresponding 95% predictive intervals. Stars show significant effect sizes when values of predictive intervals are entirely negatives.
**Figure S12:** Attribution of disturbances. The panels display the predicted coral cover across the range of disturbance values, from the minimum to the maximum values observed in American Samoa. Note that values of Heat Stress are expressed in Degree Heating Weeks and Cyclone Exposure in hours. The lines correspond to the averaged predicted values and shaded areas their associated 95% prediction intervals. Effects have been calculated at the mean values of all the other covariates.
**Figure S13:** Spatial predictions of coral cover under fixed low and high levels of cyclone exposure.
**Figure S14:** Associated relative changes of coral cover between low and high levels of cyclone exposure.
**Figure S15:** Model goodness‐of‐fit for the American Samoa region. Coral cover observations at data‐cells were used to fit the spatio‐temporal models. The R2 statistic measures how well the predicted values reproduce the observations, with values closer to 1 indicating a better fit along the 1:1 red dotted line.
**Figure S16:** QQ plot residuals, dispersion and deviation diagnostics.

## Data Availability

Appendices and associated code required to reproduce the results can be accessed at https://github.com/open‐AIMS/RC_modelling. The data used in use cases are available at Australian Institute of Marine Science (AIMS) (2025).
