# Supplementary material for: Predicting Coral Cover Trends From Local to Broad Spatial Scales
Source: Glob Chang Biol. 2026 Aug 5;32(8):e70998. doi: 10.1111/gcb.70998 (PMC13441547; doi:10.1111/gcb.70998)
Supplement: Supplementary file 1 [file GCB-32-e70998-s001.html]

Appendix B


# Appendix B

USE CASE: CENTRAL GREAT BARRIER REEF

Authors

Affiliations

Julie Vercelloni

Australian Institute of Marine Science, Cape Cleveland, 4810, Queensland, Australia

Centre for Data Science, Queensland University of Technology, Brisbane 4000, Queensland, Australia

Murray Logan

Australian Institute of Marine Science, Cape Cleveland, 4810, Queensland, Australia

Andrew Zammit‐Mangion

School of Mathematics and Statistics, University of New South Wales, Sydney 2033, New South Wales, Australia

Securing Antarctica’s Environmental Future, University of Wollongong, Wollongong 2522, New South Wales, Australia

Matthew Sainsbury‐Dale

Statistics Program, Computer, Electrical and Mathematical Sciences and Engineering Division, King Abdullah University of Science and Technology, Thuwal 23955 – 6900, Saudi Arabia

Britta Schaffelke

Australian Institute of Marine Science, Cape Cleveland, 4810, Queensland, Australia

Kerrie Mengersen

Centre for Data Science, Queensland University of Technology, Brisbane 4000, Queensland, Australia

Manuel González‐Rivero

Australian Institute of Marine Science, Cape Cleveland, 4810, Queensland, Australia

## Table of contents

- The Central Great Barrier Reef
  - Wet Tropics
  - Burdekin
  - Mackay Whitsunday
- Model predictions
  - Wet Tropics
  - Burdekin
  - Mackay Whitsunday
- Association with Disturbances
  - Wet Tropics
  - Burdekin
  - Mackay Whitsunday
- Model diagnostics
  - Model goodness-of-fit
  - Model residuals
  - Leave-out-data analysis
  - Basis function exploratory analysis

Important

To be displayed on a big screen.

## The Central Great Barrier Reef

The Australian Great Barrier Reef is divided into three Marine Ecoregions of the World (MEOWs), corresponding to its northern, central, and southern regions. In this case study, we focus on the central region (15.4–20.7° S), which spans approximately 5,300 km². Due to its large spatial extent, the central GBR is further subdivided into three Natural Resource Management (NRM) subregions (Figure 1). These subregions do not fully align with the regional boundaries: the Wet Tropics and Burdekin subregions fall entirely within the central region, whereas only 58.3% of the Mackay Whitsunday subregion is included.

Coral cover observations were extracted from the ReefCloud database on 30 July 2025. A total of 1728 transects from 358 coral reefs collected between 2006 and 2024, across five monitoring programs. The predictive model was fitted at the scale of the subregions, resulting in three individual models.

Figure 1: Locations of the three subregions along the Great Barrier Reef. Red dots indicates the predictive cells containing coral cover observations between 2006-2024.

### Wet Tropics

The Wet Tropics subregion encompasses an estimated 1,441 km² of reef area with 7.1% of the cells containing data from 220 coral reefs and 856 transects. Five monitoring programs were established in the subregion with varying temporal resolutions (Table 1). At the local scale, observed coral cover varied from 2.21% to 74.6% with a median coral cover values estimated at 23.76% between 2006 to 2024 (Figure 2).

The extracted disturbance values revealed multiple environmental events, including four years with non-zero Degree Heating Weeks (DHW) values and eight years with detected exposure to cyclonic waves (Figure 3). Despite covering only a small portion of the subregion, predictive cells with data (data-cells) captured disturbance intensity that reflected the subregional signal. Although, the 2017 and 2024 heat stress event and, 2023 cyclone were more severe within the data-cells compared to the subregional mean.

Table 1: Summary of public Wet Tropics datasets available in the ReefCloud pipeline as of December 2025. Percentages are based on the absolute counts provided in parentheses. Monitoring programs are classified by temporal resolution with short-term (0–5 years), medium-term (6–15 years), and long-term (>15 years). Data are considered public when stakeholders have consented to include them in analytical analyses.

| Program code | No. of reefs | No. of transects | Short-term data (%) | Medium-term data (%) | Long-term data (%) |
| --- | --- | --- | --- | --- | --- |
| 1492 | 24.5% (54) | 23.8% (204) | 98.5% (201) | 1.5% (3) | 0% (0) |
| 1979 | 24.5% (54) | 31.5% (270) | 0% (0) | 100% (270) | 0% (0) |
| 246 | 30% (66) | 38.6% (330) | 18.2% (60) | 12.1% (40) | 69.7% (230) |
| 47 | 9.5% (21) | 3.2% (27) | 88.9% (24) | 11.1% (3) | 0% (0) |
| 511 | 11.4% (25) | 2.9% (25) | 100% (25) | 0% (0) | 0% (0) |

Figure 2: Temporal pattern of mean coral cover (%) by data‐cells in the Wet Tropics (2006–2024). White gaps represent missing data.

Figure 3: Proportion of cells affected by disturbances for all-cells and data‐cells for each year between 2006 and 2024 (in the case of data‐cells, the proportion is calculated with respect to the number of all‐cells). Colours indicate a) average Degree Heating Weeks (DHW) and b) average exposure to cyclone waves.

Figure 4: Correlation matrix of cyclone-exposure and heat-stress predictors at lags 0, 1, and 2 years. Numbers indicate Pearson correlation coefficients calculated using pairwise complete observations, and variables are ordered using hierarchical clustering with highly correlated variables grouped together. The figure was used to identify highly collinear predictors (|r| > 0.7) and inform model variable selection.

### Burdekin

Burdekin subregion encompasses an estimated 1507 km² of reef area with 4.2% of the predictive cells containing data from 178 coral reefs and 496 transects. The temporal resolutions of data collected in this subregion were similar to the Wet Tropics (Table 2). At local scale, coral cover varied from 1.1% to 80.3% with an average coral cover estimated at 20.9% between 2007-2024 (Figure 5).

We detected seven years with non-zero DHW values and nine years with detected exposure to cyclonic waves, exceeding the number of disturbance events observed in the Wet Tropics (Figure 6). Mean DHW values recorded at the data-cells level tended to be higher than regional means in 2017, 2020, 2022 and 2024. In contrast, cyclone exposure showed the opposite pattern, with mean values generally lower at the data-cells level than across all predictive cells.

Table 2: Summary of public Burdekin datasets available in the ReefCloud pipeline as of December 2025. Percentages are based on the absolute counts provided in parentheses. Monitoring programs are classified by temporal resolution with short-term (0–5 years), medium-term (6–15 years), and long-term (>15 years). Data are considered public when stakeholders have consented to include them in analytical analyses.

| Program code | No. of reefs | No. of transects | Short-term data (%) | Medium-term data (%) | Long-term data (%) |
| --- | --- | --- | --- | --- | --- |
| 1492 | 30.3% (54) | 10.9% (54) | 61.1% (33) | 38.9% (21) | 0% (0) |
| 1979 | 32% (57) | 57.5% (285) | 0% (0) | 100% (285) | 0% (0) |
| 246 | 12.4% (22) | 22.2% (110) | 0% (0) | 9.1% (10) | 90.9% (100) |
| 47 | 5.6% (10) | 2% (10) | 100% (10) | 0% (0) | 0% (0) |
| 511 | 19.7% (35) | 7.5% (37) | 100% (37) | 0% (0) | 0% (0) |

Figure 5: Temporal pattern of mean coral cover (%) by data‐cells in Burdekin (2007–2024). White gaps represent missing data.

Figure 6: Proportion of cells affected by disturbances for all-cells and data‐cells for each year between 2007 and 2024 (in the case of data‐cells, the proportion is calculated with respect to the number of all‐cells). Colours indicate a) average Degree Heating Weeks (DHW) and b) average exposure to cyclone waves.

Figure 7: Correlation matrix of cyclone-exposure and heat-stress predictors at lags 0, 1, and 2 years. Numbers indicate Pearson correlation coefficients calculated using pairwise complete observations, and variables are ordered using hierarchical clustering with highly correlated variables grouped together. The figure was used to identify highly collinear predictors (|r| > 0.7) and inform model variable selection.

### Mackay Whitsunday

Mackay Whitsunday subregion encompasses an estimated 2601 km² of reef area with 1.3% of the cells containing data from 94 coral reefs and 338 transects. Three monitoring programs were present in the subregion with mainly medium- and long-term data (Table 3). At the local scale, coral cover values varied from a minimum of 1.8% to a maximum of 67.8% between 2007 and 2024 with a regional average of 24.8% (Figure 8).

This subregion experienced the highest number of recorded cyclone events (nine) compared to the two other subregions, with a maximum mean cyclone exposure estimated at 42 hours of wave exposure associated with cyclone Debbie (Figure 9).

Table 3: Summary of public Mackay Whitsunday datasets available in the ReefCloud pipeline as of December 2025. Percentages are based on the absolute counts provided in parentheses. Monitoring programs are classified by temporal resolution with short-term (0–5 years), medium-term (6–15 years), and long-term (>15 years). Data are considered public when stakeholders have consented to include them in analytical analyses.

| Program code | No. of reefs | No. of transects | Short-term data (%) | Medium-term data (%) | Long-term data (%) |
| --- | --- | --- | --- | --- | --- |
| 1492 | 35.1% (33) | 9.8% (33) | 36.4% (12) | 63.6% (21) | 0% (0) |
| 1979 | 35.1% (33) | 48.8% (165) | 0% (0) | 100% (165) | 0% (0) |
| 246 | 29.8% (28) | 41.4% (140) | 0% (0) | 0% (0) | 100% (140) |

Figure 8: Temporal pattern of mean coral cover (%) by data‐cells in Mackay Whitsunday (2007–2024). White gaps represent missing data.

Figure 9: Proportion of cells affected by disturbances for all-cells and data‐cells for each year between 2007 and 2024 (in the case of data‐cells, the proportion is calculated with respect to the number of all‐cells). Colours indicate a) average Degree Heating Weeks (DHW) and b) average exposure to cyclone waves.

Figure 10: Correlation matrix of cyclone-exposure and heat-stress predictors at lags 0, 1, and 2 years. Numbers indicate Pearson correlation coefficients calculated using pairwise complete observations, and variables are ordered using hierarchical clustering with highly correlated variables grouped together. The figure was used to identify highly collinear predictors (|r| > 0.7) and inform model variable selection.

## Model predictions

### Wet Tropics

At the local scale, predicted coral cover ranged from 2.7% [0.5–7.1%, 95% predictive intervals] to 67% [57.5–75.3%] (Figure 11). Between 2006 and 2012, predicted coral cover was generally low in the southern section of the Wet Tropics subregion. However, the coral cover increased in these locations between 2013 and 2016. The impact of the 2017 heat stress event is evident in these locations, with consistently lower predicted coral cover from 2018 to 2024 compared to the other sections. Coral cover values show a subregion-wide recovery in 2023 and 2024, although some locations in the western part exhibited high predicted values across the entire surveyed year range. The spatio-temporal patterns of uncertainty range reveal lower uncertainty at and near data-cell locations, with increasing uncertainty in areas farther from these sites, expected and consistent throughout the years (Figure 12). Additionally, uncertainty range increased across the region in 2023 and 2024.

Figure 11: Predicted coral cover values across the Wet Tropics for each year between 2006-2024. Values correspond to the posterior means.

Figure 12: Uncertainty associated with predictions across the Wet Tropics for each year between 2006-2024. Values correspond to the range between the lower and upper bounds of the 95% posterior predictive intervals of the estimated coral cover.

### Burdekin

In Burdekin, a north–south gradient in coral cover was observed from 2007 to 2014, with higher values in the southern part of the region compared to the northern part (Figure 13). Subregional recovery occurred through 2015 and 2018, followed by a broad decline in 2019 potentially associated with the 2018 cyclone and recovery in 2020. Then patchy declines in coral cover were predicted across the region up to 2024. Uncertainty range remained high in the southern sector throughout most years, decreasing by 2024 (Figure 14).

Figure 13: Predicted coral cover values across Burdekin for each year between 2007-2024. Values correspond to the posterior means.

Figure 14: Uncertainty associated with predictions across Burdekin for each year between 2007-2024. Values correspond to the range between the lower and upper bounds of the 95% posterior predictive intervals of the estimated coral cover.

### Mackay Whitsunday

Model predictions were relatively homogeneous across the Mackay-Whitsunday subregion from 2007 to 2014. From 2015 to 2018, increases in coral cover were predicted primarily in the northern and eastern parts of the subregion (Figure 15). Uncertainty range remained low to moderate until 2016. From 2017 onward, uncertainty range increased across most of the subregion (Figure 16).

Figure 15: Predicted coral cover values across Mackay Whitsunday for each year between 2007-2024. Values correspond to the posterior means.

Figure 16: Uncertainty associated with predictions across Mackay Whitsunday for each year between 2007-2024. Values correspond to the range between the lower and upper bounds of the 95% posterior predictive intervals of the estimated coral cover.

## Association with Disturbances

### Wet Tropics

In the Wet Tropics, cyclone exposure at lags 0 and 1 years, as well as heat stress across all time lags were negatively associated with subregional coral cover change, with 95% prediction intervals not overlapping zero (Figure 3 in the manuscript). The largest absolute difference between the minimum and maximum observed disturbance levels occurred for cyclone exposure at lag 1, corresponding to a 37.6% reduction in coral cover (95% prediction interval: −41.0% to −3.1%, Figure 17). Heat stress was associated with an 18.3% reduction in coral cover (−23.3% to −11.5%), and this difference declined with increasing lag: 15.1% at lag 1 (−20.7% to −7.0%) and 7.4% at lag 2 (−11.6% to −0.5%).

Spatially, higher DHW alone (i.e., excluding other disturbances and time-lag effects) was associated with subregional differences in coral cover (Figure 18), with absolute differences estimated to range from 3.5% to 10.9%, and a subregional average difference of 7.0% (Figure 19).

Figure 17: Association with Disturbances. The panels display the predicted coral cover across the range of disturbance values, from the minimum to the maximum values observed in Wet Tropics. Note that values of Heat Stress are expressed in Degree Heating Weeks and Cyclone Exposure in hours. The lines correspond to the averaged predicted values and shaded areas their associated 95% prediction intervals. Effects have been calculated at the mean values of all the other covariates.

Figure 18: Spatial predictions of coral cover under fixed low and high levels of heat stress across the Wet Tropics.

Figure 19: Associated absolute changes of coral cover between low and high levels of heat stress across the Wet Tropics.

### Burdekin

In the Burdekin subregion, a negative association was detected only for cyclone exposure at lag 1 year (Figure 3 in the manuscript). This disturbance was associated with coral cover decline of 42.6% between the minimum and maximum observed cyclone exposure values (95% prediction interval: −43.3% to −33.3%, Figure 20).

Higher exposure to cyclonic waves from 0 to 30 hours (Figure 21) was associated with varying subregional differences in coral cover, ranging from 3.2% to 24.3%, with an average absolute decline of 13.6% (Figure 22). Most subregional estimates (75% percentile) fell between 11.9% and 24.3%, indicating that the majority of areas experienced moderate to large declines.

Figure 20: Association with Disturbances. The panels display the predicted coral cover across the range of disturbance values, from the minimum to the maximum values observed in Burdekin. Note that values of Heat Stress are expressed in Degree Heating Weeks and Cyclone Exposure in hours. The lines correspond to the averaged predicted values and shaded areas their associated 95 % prediction intervals. Effects have been calculated at the mean values of all the other covariates.

Figure 21: Spatial predictions of coral cover under fixed low and high levels of cyclone exposure at lag 1 year across Burdekin.

Figure 22: Associated absolute changes of coral cover between low and high levels of cyclone exposure at lag 1 year across Burdekin.

### Mackay Whitsunday

In the Mackay Whitsunday subregion, cyclone exposure was negatively associated with subregional coral cover across all time lags and heat stress at lag 1 year (Figure 3 in the manuscript). The largest absolute difference between the minimum and maximum observed cyclone exposure levels was estimated at 43.8% at lag 0 (95% prediction interval: −40.1% to −37.6%), followed by 42.1% at lag 2 (−39.3% to −29.0%) and 35.1% at lag 1 (−36.9% to −16.3%, Figure 23). Heat stress at lag 1 year was associated with a smaller decline of 11.0%, with large uncertainty (−18.9% to -1.5%).

Comparing predictions under no cyclonic wave exposure at lag 1 year with those under high exposure across space (Figure 24) showed large absolute declines in the northern section of the region (Figure 25), with coral cover losses reaching up to 19.9% and a subregional average decline of 11.2%.

Figure 23: Association with Disturbances. The panels display the predicted coral cover across the range of disturbance values, from the minimum to the maximum values observed in Mackay Whitsunday. Note that values of Heat Stress are expressed in Degree Heating Weeks and Cyclone Exposure in hours. The lines correspond to the averaged predicted values and shaded areas their associated 95 % prediction intervals. Effects have been calculated at the mean values of all the other covariates.

Figure 24: Spatial predictions of coral cover under fixed low and high levels of cyclone exposure across Mackay Whitsunday.

Figure 25: Associated absolute changes of coral cover between low and high levels of cyclone exposure across Mackay Whitsunday.

## Model diagnostics

### Model goodness-of-fit

Model performance showed a strong agreement between predicted and observed coral cover at the data-tier level, with an \(R^2\) scores ranging from 79% for Burdekin up to 89% for the Wet Tropics (Figure 26) with normally distributed residuals and absences of overdispersions (Figure 27).

Figure 26: Model goodness-of-fit for the three subregions. Coral cover observations at the local scale were used to fit the spatio-temporal models. The R² statistic measures how well the predicted values reproduce the observations, with values closer to 1 indicating a better fit along the 1:1 red dotted line.

### Model residuals

Figure 27: QQ plot residuals, dispersion and deviation diagnostics.

### Leave-out-data analysis

This analysis was performed on the Wet Tropics subregion only.

The leave-out data approach is used to evaluate the influence of specific observations using prediction-performance measures. The full model is fitted on a train dataset composed of a random sample of observations and prediction-performance measures are computed on the leave-out observations.

We found that the predictive performance of the spatio-temporal model is more sensitive to the number of monitoring locations, with accuracy declining when sites or reefs are removed from the dataset (Figure 28). In contrast, model performance is less affected by the number of replicated years.

Five validation tests are developed:

- 1. rm(20% obs): 20% of data were randomly removed without structure.
- 2. rm(20% reef): 20% of reefs were randomly removed.
- 3. rm(20% site): 20% of sites were randomly removed.
- 4. rm(20% transect): 20% of transects were randomly removed.
- 5. rm(3YRS): 3 years of observations were removed within each location (locations with less than 4 years of data were not used).

Four predictive measures are used:

- 95% coverage interval (CvgErr): evaluates how often predictions include true observations, with the goal of capturing the true values 95% of the time. It is estimated as follows:

\[
\text{CvgErr}(z, \ell, u) \;=\;
\left| 0.95 \;-\; \frac{1}{n} \sum\_{i=1}^{n} \mathbf{1}\!\left( \ell\_i < z\_i < u\_i \right) \right|
\]

where \(z = \{z\_1, z\_2, \dots, z\_n\}\) are the coral cover observations, \(\ell\) and \(u\) are the lower and upper bounds of the predictive intervals, \(n\) the total number of predictions, and \(\mathbf{1}(\cdot)\) is the indicator function (1 if the condition is true, 0 otherwise).

- 95% interval score (IS): rewards prediction intervals that include the true observations (accuracy) and penalizes those that are too narrow or too wide (precision). It is computed as follow:

\[
\text{IS}\_{95} \;=\; \frac{1}{n} \sum\_{i=1}^{n}
\Bigg[
(u\_i - \ell\_i)
+ \frac{2}{\alpha} (\ell\_i - y\_i)\,\mathbf{1}(y\_i < \ell\_i)
+ \frac{2}{\alpha} (y\_i - u\_i)\,\mathbf{1}(y\_i > u\_i)
\Bigg]
\]

where \(\alpha = 0.05\), \(\ell\) and \(u\) are the lower and upper bounds of the predictive intervals, \(n\) the total number of predictions, and \(y\) are observed coral cover.

- Root-mean-squared prediction error (RMSPE) - how far off model predictions are from true observations without considering for uncertainty.

\[
\text{RMSPE} \;=\; \sqrt{ \frac{1}{n} \sum\_{i=1}^{n} (y\_i - \hat{y}\_i)^2 }
\]

where \(y\) and \(\hat{y}\) are the observed and predicted coral cover values, respectively, and \(n\) the total number of observations.

- Continuous Ranked Probability Score (CRPS) - represents the quality of the predictions over the entire predictive probability distribution penalizing predictions that are inaccurate, imprecise or overconfident.

\[
\text{CRPS}(F, y) \;=\; \sigma \left[
z \left( 2 \Phi(z) - 1 \right) \;+\; 2 \,\phi(z) \;-\; \frac{1}{\sqrt{\pi}}
\right],
\quad z = \frac{y - \mu}{\sigma}
\]

where \(y\) is the observed coral cover values, \(\mu\) and \(\sigma\) are the mean and the standard deviation of the predictive normal distribution, \(\phi(.)\) represented the standard normal probability density function and \(\Phi\) the cumulative distribution function.

These predictive measures give a single number with low scores representing better performances.

Results from this analysis indicate that the model predictive performance declines when entire blocks of observations, such as specific reefs and sites, are removed (Figure 28).

Figure 28: Metrics of model predictive performance by tests. Each axis represents one of the evaluation metrics described above. The length of each arm represents the model predictive performance on that metric for a given test, with values scaled from 1 (poor) to 0 (best). Overall, better predictive performances of the models are indicated by smaller polygons.

### Basis function exploratory analysis

The aim of this analysis is to explore the influence of the basis function formulation with a focus on the temporal dimension. To do this, we compare four model performance using different number of basis functions in the temporal basis function:

- Full: number and location of temporal basis functions automatically estimated from the FRK function and adopted by ReefCloud.
- 5: five temporal basis functions randomly selected from the FRK auto basis function.
- 3: three temporal basis functions randomly selected from the FRK auto basis function.
- 1: one temporal basis function randomly selected from the FRK auto basis function.

As anticipated, the number of temporal basis functions in the model affects the smoothness of coral trends at the tier level, which subsequently impacts the uncertainty in regional trends (Figure 30). The number of basis functions also influence the pattern of attribution of disturbance effects that is inferred when fitting the model (Figure 31).

Figure 29: Locations of the basis functions for each model with a) Default, b) 5, c) 3 and d) 1.

Figure 30: Regional trends predicted by the spatio-temporal models. Solid lines represent mean predicted coral cover, with shaded areas indicating predictive intervals.

Figure 31: Estimated effect sizes of disturbance effects. The points represent the estimated effect and the intervals represent the corresponding 95% confidence intervals.
