# Supplementary material for: Predicting Coral Cover Trends From Local to Broad Spatial Scales
Source: Glob Chang Biol. 2026 Aug 5;32(8):e70998. doi: 10.1111/gcb.70998 (PMC13441547; doi:10.1111/gcb.70998)
Supplement: Supplementary file 2 [file GCB-32-e70998-s002.html]

Appendix C


# Appendix C

USE CASE: AMERICAN SAMOA

Authors

Affiliations

Julie Vercelloni

Australian Institute of Marine Science, Cape Cleveland, 4810, Queensland, Australia

Centre for Data Science, Queensland University of Technology, Brisbane 4000, Queensland, Australia

Murray Logan

Australian Institute of Marine Science, Cape Cleveland, 4810, Queensland, Australia

Andrew Zammit‐Mangion

School of Mathematics and Statistics, University of New South Wales, Sydney 2033, New South Wales, Australia

Securing Antarctica’s Environmental Future, University of Wollongong, Wollongong 2522, New South Wales, Australia

Matthew Sainsbury‐Dale

Statistics Program, Computer, Electrical and Mathematical Sciences and Engineering Division, King Abdullah University of Science and Technology, Thuwal 23955 – 6900, Saudi Arabia

Britta Schaffelke

Australian Institute of Marine Science, Cape Cleveland, 4810, Queensland, Australia

Kerrie Mengersen

Centre for Data Science, Queensland University of Technology, Brisbane 4000, Queensland, Australia

Manuel González‐Rivero

Australian Institute of Marine Science, Cape Cleveland, 4810, Queensland, Australia

## Table of contents

- American Samoa
- Model predictions
  - Tutuila
  - Ofu-Olosega
  - Ta‘ū
  - Rose Atoll
  - Swains Island
- Association with Disturbances
- Model diagnostics
  - Model goodness-of-fit
  - Model residuals

Important

To be displayed on a big screen.

## American Samoa

American Samoa comprises three islands and two atolls, representing a total reef area of 59.2 km², with 75% of predictive cells containing data. This case study focuses on islands and atolls (hereafter referred to as subregions) belonging to the single Marine Ecoregion of the World (MEOW) that encompasses the entire country (Figure 1) and its subregions (Figure 2).

Coral cover observations were extracted from the ReefCloud database on 15 March 2026. A total of 507 transects from 507 sites collected between 2015 and 2023 by the NOAA’s U.S. Pacific Islands National Coral Reef Monitoring Program (Table 1) at depths varying 3 and 10 metres. At the local scale, observed coral cover varied from 0.5% to 69.6% with a median coral cover values estimated at 25.3% between 2015 to 2023 (Figure 3).

American Samoa was exposed to five heat-stress events and two cyclones between 2015 and 2023 (Figure 4). Disturbance events were infrequently captured in the data cells: only the 2015 heat-stress event was recorded at higher intensity to the regional signal, and Cyclone Gita (2018) at a comparable intensity to the regional signal.

Figure 1: Locations of the five islands across the American Samoa. Red dots indicates the data cells containing coral cover observations between 2015-2023.

Figure 2: Zoom-in on the subregions. Red dots indicates the data cells containing coral cover observations between 2015-2023.

Table 1: Summary of public American Samoa datasets available in the ReefCloud pipeline as of March 2026. Percentages are based on the absolute counts provided in parentheses. Monitoring programs are classified by temporal resolution with short-term (0–5 years), medium-term (6–15 years), and long-term (>15 years). Data are considered public when stakeholders have consented to include them in analytical analyses.

| Program.code | No. of sites | No. of transects | Short-term data (%) | Medium-term data (%) | Long-term data (%) |
| --- | --- | --- | --- | --- | --- |
| 56 | 100% (507) | 100% (507) | 100% (507) | 0% (0) | 0% (0) |

Figure 3: Temporal pattern of mean coral cover (%) by data cells in the American Samoa (2015–2023). White gaps represent missing data.

Figure 4: Proportion of regional areas affected by disturbances across two data levels all-cells and data-cells for each year between 2015 and 2023 (in the case of data-cells, the proportion is calculated with respect to the number of all-cells). Colours indicate a) average Degree Heating Weeks (DHW) and b) average exposure to cyclone waves.

Figure 5: Correlation matrix of cyclone-exposure and heat-stress predictors at lag 0 years. Numbers indicate Pearson correlation coefficients calculated using pairwise complete observations, and variables are ordered using hierarchical clustering with highly correlated variables grouped together. The figure was used to identify highly collinear predictors (|r| > 0.7) and inform model variable selection.

## Model predictions

### Tutuila

Figure 6: a) Predicted coral cover values across Tutuila subregion and b) associated uncertainty range for each year between 2015-2023. Values correspond to their psoterior means and the lower and upper bounds of the 95% posterior credible intervals, respectively.

### Ofu-Olosega

Figure 7: a) Predicted coral cover values across Ofu-Olosega subregion and b) associated uncertainty range for each year between 2015-2023. Values correspond to their psoterior means and the lower and upper bounds of the 95% posterior credible intervals, respectively.

### Ta‘ū

Figure 8: a) Predicted coral cover values across Ta‘ū subregion and b) associated uncertainty range for each year between 2015-2023. Values correspond to their psoterior means and the lower and upper bounds of the 95% posterior credible intervals, respectively.

### Rose Atoll

Figure 9: a) Predicted coral cover values across Rose Atoll subregion and b) associated uncertainty range for each year between 2015-2023. Values correspond to their psoterior means and the lower and upper bounds of the 95% posterior credible intervals, respectively.

### Swains Island

Figure 10: a) Predicted coral cover values across Swains Island subregion and b) associated uncertainty range for each year between 2015-2023. Values correspond to their psoterior means and the lower and upper bounds of the 95% posterior credible intervals, respectively.

## Association with Disturbances

In American Samoa, cyclone exposure was negatively associated with coral cover change, with 95% prediction intervals not overlapping zero (Figure 11). The absolute decline between the minimum and maximum observed cyclone exposure, corresponds to a 16.1% reduction in coral cover (95% prediction interval: −27.8% to -1.2%, Figure 12).

Spatially, increasing magnitude of cyclone exposure alone (i.e., excluding heat stress) contributed to regional declines in coral cover (Figure 13), with absolute losses estimated to range from 4.1% to 27.0%, and a regional average decline of 15.8% (Figure 14).

Figure 11: Predicted effect sizes of disturbances. The dots represent the predicted averaged effect and the intervals represent the corresponding 95% predictive intervals. Stars show significant effect sizes when values of predictive intervals are entirely negatives.

Figure 12: Attribution of disturbances. The panels display the predicted coral cover across the range of disturbance values, from the minimum to the maximum values observed in American Samoa. Note that values of Heat Stress are expressed in Degree Heating Weeks and Cyclone Exposure in hours. The lines correspond to the averaged predicted values and shaded areas their associated 95 % prediction intervals. Effects have been calculated at the mean values of all the other covariates.

Figure 13: Spatial predictions of coral cover under fixed low and high levels of cyclone exposure.

Figure 14: Associated relative changes of coral cover between low and high levels of cyclone exposure.

## Model diagnostics

### Model goodness-of-fit

Figure 15: Model goodness-of-fit for the American Samoa region. Coral cover observations at data-cells were used to fit the spatio-temporal models. The R² statistic measures how well the predicted values reproduce the observations, with values closer to 1 indicating a better fit along the 1:1 red dotted line.

### Model residuals

Figure 16: QQ plot residuals, dispersion and deviation diagnostics.
