# Supplementary material for: Predicting Coral Cover Trends From Local to Broad Spatial Scales
Source: Glob Chang Biol. 2026 Aug 5;32(8):e70998. doi: 10.1111/gcb.70998 (PMC13441547; doi:10.1111/gcb.70998)
Supplement: Supplementary file 3 [file GCB-32-e70998-s003.html]

Appendix A


# Appendix A

USE CASE: SIMULATION EXPERIMENTS

Authors

Affiliations

Julie Vercelloni

Australian Institute of Marine Science, Cape Cleveland, 4810, Queensland, Australia

Centre for Data Science, Queensland University of Technology, Brisbane 4000, Queensland, Australia

Murray Logan

Australian Institute of Marine Science, Cape Cleveland, 4810, Queensland, Australia

Andrew Zammit‐Mangion

School of Mathematics and Statistics, University of New South Wales, Sydney 2033, New South Wales, Australia

Securing Antarctica’s Environmental Future, University of Wollongong, Wollongong 2522, New South Wales, Australia

Matthew Sainsbury‐Dale

Statistics Program, Computer, Electrical and Mathematical Sciences and Engineering Division, King Abdullah University of Science and Technology, Thuwal 23955 – 6900, Saudi Arabia

Britta Schaffelke

Australian Institute of Marine Science, Cape Cleveland, 4810, Queensland, Australia

Kerrie Mengersen

Centre for Data Science, Queensland University of Technology, Brisbane 4000, Queensland, Australia

Manuel González‐Rivero

Australian Institute of Marine Science, Cape Cleveland, 4810, Queensland, Australia

## Table of contents

- Summary of the ReefCloud database
- The synthetic study
  - Synthos
- Simulation Scenarios
- Predictive Performances
- Monitoring Locations
- Benthic Monitoring Data
- Domain-level Trends
- Model Prediction
- Model Uncertainty
- Trends at Local Scale
- Association with disturbances
- Model fit
- Cyclone
- Heat stress
- Details on predictive measures

Important

To be displayed on a big screen.

## Summary of the ReefCloud database

In April 2026, the ReefCloud database contained 4,722 publicly monitored reef locations spanning 51 countries across four regions (Table 1). Most regions were dominated by short-term monitoring (sites re-surveyed every 1–5 years), while medium-term monitoring (5–15 years) was rare. Long-term monitoring (>15 years) was observed only in Australia. Most of monitoring locations were concentrated in Australia (42.1%) and in the Pacific Ocean excluding Australia (32.4%).

These characteristics highlight both the dominance of short-term public datasets and the geographic concentration of monitoring efforts within the ReefCloud database.

Table 1: Summary of public datasets available in the ReefCloud pipeline as of April 2026. Percentages are based on the absolute counts provided in parentheses. Monitoring programs are classified by temporal resolution with short-term (0–5 years), medium-term (6–15 years), and long-term (>15 years). Data are considered public when stakeholders have consented to include them in analytical analyses. Regions were extracted using the geospatial data of (British Oceanographic Data Centre (BODC) 2023).

| region | No. of sites | No. of countries | No. of projects | Short-term data | Medium-term data | Long-term data |
| --- | --- | --- | --- | --- | --- | --- |
| ATLANTIC OCEAN | 5.3% (252) | 25.5% (13) | 10.4% (58) | 100% (252) | 0% (0) | 0% (0) |
| AUSTRALIA | 36.4% (1721) | 2% (1) | 29.8% (167) | 92.2% (1587) | 4.7% (80) | 3.1% (54) |
| INDIAN OCEAN (excl. Australia) | 14.8% (698) | 25.5% (13) | 14.6% (82) | 99.9% (697) | 0.1% (1) | 0% (0) |
| PACIFIC OCEAN (excl. Australia) | 31.7% (1497) | 31.4% (16) | 27% (151) | 97.9% (1466) | 2.1% (31) | 0% (0) |
| SOUTH CHINA AND EASTERN ARCHIPELAGIC SEAS | 11.8% (558) | 15.7% (8) | 18.2% (102) | 99.3% (554) | 0.7% (4) | 0% (0) |

## The synthetic study

We developed a simulator to assess the robustness of our predictive model under controlled conditions, where simulated coral cover values are available across the entire spatial domain. In the statistical modelling framework, all data are used to fit the model which limits the ability to reserve truly independant observations for validation (McElreath 2018), although leave-out-data analyses can still be implemented (see Appendix A). This synthetic study allowed us to evaluate prediction accuracy across the full predictive domain, rather than at a limited number of randomly chosen locations. Such simulation-based approaches are commonly used to assess analytical methods and are widely applied in ecology and statistics (Zurell et al. 2010). We also used to approach to test the predictive model under varying spatial and temporal data resolutions as examplied in Table 1.

### Synthos

The R package synthos GitHub generates synthetic data with the primarly aim to test the model robstuness under varying spatial and temporal data resolutions, in a controlled enviroment. The data are created from spatio-temporal dependency structures mimicing baselines, population dynamics, disturbance regimes and stochastic processes.

The generation of synthetic data is based on four main steps:

- Generation of the spatio-temporal domains: the first step consists to generate virtual coral reefs across a spatial domain.
- Generation of the disturbances: two types of disturbance are generated (Heat Stress events, Cyclones) using a mechanistic approach that generates their own spatial and temporal patterns across the spatial domain.
- Generation of baselines: representing values in coral cover the year prior the first sampling.
- Generation of sampling designs: selection of the number of reef monitoring locations and surveyed years.

Coral cover values are generated by combining baseline values, disturbance effects, and growth rates, and then projecting their cumulative effects across the spatial domain. The sampling design specifies the number of observations and years to be selected within a domain, creating the observed dataset. Models are then fitted to these observed data, and their predictive performance is evaluated at locations that are not monitored but for which coral cover values are known. Indeed, in our synthetic framework, the underlying data-generating process is known, and the corresponding simulated values can therefore be treated as the true reference against which model performance is evaluated.

## Simulation Scenarios

Three scenarios were tested, representing short-term, medium-term, long-term, and temporally sparse annual monitoring observations, each based on 15 permanent reefs (Table 2). This sample size corresponds to one-third of the total number of simulated reefs. A fourth scenario represents a sparse temporal resolution, in which 50 reefs are monitored over 15 years with varying survey intensities. Each reef is randomly surveyed between 2 and 15 times, with no more than 5 years between any two consecutive surveys. This scenario mimics the aggregation of different data sources with permanent and opportunistic observations.

Table 2: Details of the simulation scenarios.

| Scenario | No. of reefs | No. of years |
| --- | --- | --- |
| Short-term | 15 | 3 |
| Medium-term | 15 | 12 |
| Long-term | 15 | 20 |
| Temporally-sparse | 15 | 2-15 |

Other settings were kept constant across the simulation scenarios. The coral baseline was generated using a west–east gradient of coral cover. The relative influence of disturbances on coral dynamics was set to 60% for heat stress events and 40% for cyclones. Coral growth was fixed at 30%, with a possible coverage range from 1% to 70%.

The hierarchical sampling design within reef incorporated a nested structure of 1 depth (10m), 2 permanent sites, 5 transects per site, 100 photoquadrats per transect. Finally, point-based observations are generated with 50 points per frame. Coral cover values are summarized at the transect scale by summing the number of coral count across all photoquadrats.

The spatial domain is divided into a 0.1° grid (~10 × 10 km hexagonal units, referred to as ‘predictive cells’). Disturbance values are predicted onto these predictive cells, and transect-based coral cover values are aggregated within each predictive cell (named data-cells) for use in the spatio-temporal model.

## Predictive Performances

For each scenario, the model’ predictive performance is tested based on four predictive measures (Table 3). These metrics are calculated based on the out-of-sample model predictions where “true” values are known but were not used by the statistical model. See Section 0.15 for the mathematical details of the predictive measures.

Table 3: Details of predictive measures used to evaluate the predictive performances of the models in the simulation experiments. The metrics yield scores between 0 and 1, where values closer to 0 indicate a better predictive performance.

| Name | Details |
| --- | --- |
| Coverage Error (CvgErr) | The absolute difference between the proportion of times the 95% prediction intervals include the true values and 0.95. |
| Root-Mean-Squared Prediction Error (RMSPE) | Indicates how far predictions deviate from true observations, without accounting for uncertainty. |
| 95% Interval Score (IS) | Balances accuracy and precision by rewarding intervals that include the true value and penalizing those that are too narrow or too wide. |
| Continuous Ranked Probability Score (CRPS) | Assesses the overall quality of the predictive distribution, penalizing inaccuracies, imprecision, and overconfidence. |

Results

Our diagnostics show that the temporally-sparse scenario (50 reefs sampled with varying temporal intensities) produces the best predictive performance for all metrics (Figure 1). This result is mainly driven by the ability of the model to leverage repeated observations to characterize temporal dynamics while also accounting for spatial structure across the full prediction domain, and explicitly capture the influences of disturbances as they unfolded across space and time. The medium- and long-term scenarios exhibit similar predictive performances with reduced predictive performance based on the continuous ranked probability (CRPS) and root-mean-squared prediction error (RMSPE) and interval score (IS). This result reflects a higher model uncertainty associated with coral cover predictions at locations without observations (Figures 23 & 24). The short-term scenario clearly differs in terms of coverage error, with true values more frequently falling outside the prediction intervals, likely reflecting intervals that are too narrow (Figure 26).

Each scenario achieved a satisfactory model fit, with \(R^2\) values ranging from 0.88 for the long-term scenario to 0.99 for the temporally sparse scenario (Figure 38). At the predictive domain level, the model reproduces the true simulated temporal patterns across scenarios, with some systematic underestimation evident in the long-term and temporally sparse scenarios (Figures 14–17). This is likely attributable to the more complex temporal dynamics resulting from an increased number of disturbances over extended simulation periods (Figures 39–46). The predictive effect sizes of disturbances indicate that uncertainty decreases from the short-term to the long-term scenario. This is primarily due to the greater and more variable disturbance exposure of reef locations across years, which allows the model to attribute their effects more accurately. The predictive effect size of cyclone exposure in the temporally sparse scenario exhibits lower uncertainty compared to the long-term scenario, whereas the effect size for heat stress shows higher uncertainty.

In summary, the simulation experiments demonstrate that the predictive model recovers simulated values and temporal patterns across varying spatio-temporal data resolutions. Long-term data are associated with greater overall uncertainty but allow for more precise attribution of disturbance effects. Short-term data, in contrast, may suffer from overestimation, characterized by narrow predictive intervals and higher uncertainty in disturbance attribution. Finally, temporally sparse data provide the best balance, yielding optimal predictive accuracy. This last result demonstrates that integrating the model within the ReefCloud pipeline will facilitate the incorporation of datasets from multiple sources, thereby increasing data support and improving model robustness.

Figure 1: Performance metrics for the four simulation scenarios.

## Monitoring Locations

- Short-term
- Medium-term
- Long-term
- Temporally-sparse

[[1]]

Figure 2: Spatial distribution of the synthetic reefs. The red dots indicate monitoring locations and hexagons represent cell boundaries. A cell is considered a data-cell if it contains a red dot; otherwise, it is classified as a predictive-cell for the scenario Short-term

[[1]]

Figure 3: Spatial distribution of the synthetic reefs. The red dots indicate monitoring locations and hexagons represent cell boundaries. A cell is considered a data-cell if it contains a red dot; otherwise, it is classified as a predictive-cell for the scenario Medium-term

[[1]]

Figure 4: Spatial distribution of the synthetic reefs. The red dots indicate monitoring locations and hexagons represent cell boundaries. A cell is considered a data-cell if it contains a red dot; otherwise, it is classified as a predictive-cell for the scenario Long-term

[[1]]

Figure 5: Spatial distribution of the synthetic reefs. The red dots indicate monitoring locations and hexagons represent cell boundaries. A cell is considered a data-cell if it contains a red dot; otherwise, it is classified as a predictive-cell for the scenario Temporally-sparse

## Benthic Monitoring Data

- Short-term
- Medium-term
- Long-term
- Temporally-sparse

[[1]]

Figure 6: Simulated transect-scale coral cover values (%) within each reef, with colours representing the two sites for the scenario Short-term

[[1]]

Figure 7: Simulated transect-scale coral cover values (%) within each reef, with colours representing the two sites for the scenario Medium-term

[[1]]

Figure 8: Simulated transect-scale coral cover values (%) within each reef, with colours representing the two sites for the scenario Long-term

[[1]]

Figure 9: Simulated transect-scale coral cover values (%) within each reef, with colours representing the two sites for the scenario Temporally-sparse

- Short-term
- Medium-term
- Long-term
- Temporally-sparse

[[1]]

Figure 10: Temporal patterns of mean coral cover (%) by site within reef. White gaps show missing data for the scenario Short-term

[[1]]

Figure 11: Temporal patterns of mean coral cover (%) by site within reef. White gaps show missing data for the scenario Medium-term

[[1]]

Figure 12: Temporal patterns of mean coral cover (%) by site within reef. White gaps show missing data for the scenario Long-term

[[1]]

Figure 13: Temporal patterns of mean coral cover (%) by site within reef. White gaps show missing data for the scenario Temporally-sparse

## Domain-level Trends

- Short-term
- Medium-term
- Long-term
- Temporally-sparse

[[1]]

Figure 14: Regional trend. The black line shows mean values and green shadows 95% predictive intervals. The dotted blue line is the true simulated temporal field estimated from the Gaussian Random Markov Field values in the simulation Short-term

[[1]]

Figure 15: Regional trend. The black line shows mean values and green shadows 95% predictive intervals. The dotted blue line is the true simulated temporal field estimated from the Gaussian Random Markov Field values in the simulation Medium-term

[[1]]

Figure 16: Regional trend. The black line shows mean values and green shadows 95% predictive intervals. The dotted blue line is the true simulated temporal field estimated from the Gaussian Random Markov Field values in the simulation Long-term

[[1]]

Figure 17: Regional trend. The black line shows mean values and green shadows 95% predictive intervals. The dotted blue line is the true simulated temporal field estimated from the Gaussian Random Markov Field values in the simulation Temporally-sparse

## Model Prediction

- Short-term
- Medium-term
- Long-term
- Temporally-sparse

[[1]]

Figure 18: Predictions of coral cover (%) in Short-term

[[1]]

Figure 19: Predictions of coral cover (%) in Medium-term

[[1]]

Figure 20: Predictions of coral cover (%) in Long-term

[[1]]

Figure 21: Predictions of coral cover (%) in Temporally-sparse

## Model Uncertainty

- Short-term
- Medium-term
- Long-term
- Temporally-sparse

[[1]]

Figure 22: Uncertainty associated with predicted coral cover values in Short-term

[[1]]

Figure 23: Uncertainty associated with predicted coral cover values in Medium-term

[[1]]

Figure 24: Uncertainty associated with predicted coral cover values in Long-term

[[1]]

Figure 25: Uncertainty associated with predicted coral cover values in Temporally-sparse

## Trends at Local Scale

- Short-term
- Medium-term
- Long-term
- Temporally-sparse

[[1]]

Figure 26: Examples of estimated coral trends at the data-cells. Black lines represent mean predicted coral cover, with shaded areas indicating 95% predictive intervals. Dots show observed coral cover averaged at the cell level, which were used as the response variable in the spatio-temporal model. for the scenario Short-term

[[1]]

Figure 27: Examples of estimated coral trends at the data-cells. Black lines represent mean predicted coral cover, with shaded areas indicating 95% predictive intervals. Dots show observed coral cover averaged at the cell level, which were used as the response variable in the spatio-temporal model. for the scenario Medium-term

[[1]]

Figure 28: Examples of estimated coral trends at the data-cells. Black lines represent mean predicted coral cover, with shaded areas indicating 95% predictive intervals. Dots show observed coral cover averaged at the cell level, which were used as the response variable in the spatio-temporal model. for the scenario Long-term

[[1]]

Figure 29: Examples of estimated coral trends at the data-cells. Black lines represent mean predicted coral cover, with shaded areas indicating 95% predictive intervals. Dots show observed coral cover averaged at the cell level, which were used as the response variable in the spatio-temporal model. for the scenario Temporally-sparse

- Short-term
- Medium-term
- Long-term
- Temporally-sparse

[[1]]

Figure 30: Examples of estimated coral trends at predictive-cells. Black lines represent mean predicted coral cover, with shaded areas indicating 95% predictive intervals, for the scenario Short-term

[[1]]

Figure 31: Examples of estimated coral trends at predictive-cells. Black lines represent mean predicted coral cover, with shaded areas indicating 95% predictive intervals, for the scenario Medium-term

[[1]]

Figure 32: Examples of estimated coral trends at predictive-cells. Black lines represent mean predicted coral cover, with shaded areas indicating 95% predictive intervals, for the scenario Long-term

[[1]]

Figure 33: Examples of estimated coral trends at predictive-cells. Black lines represent mean predicted coral cover, with shaded areas indicating 95% predictive intervals, for the scenario Temporally-sparse

## Association with disturbances

- Short-term
- Medium-term
- Long-term
- Temporally-sparse

[[1]]

Figure 34: Estimated effect sizes of disturbance exposure (in the logit scale). The points represent the estimated effect and the intervals represent the corresponding 95% predictive intervals, for the scenario Short-term

[[1]]

Figure 35: Estimated effect sizes of disturbance exposure (in the logit scale). The points represent the estimated effect and the intervals represent the corresponding 95% predictive intervals, for the scenario Medium-term

[[1]]

Figure 36: Estimated effect sizes of disturbance exposure (in the logit scale). The points represent the estimated effect and the intervals represent the corresponding 95% predictive intervals, for the scenario Long-term

[[1]]

Figure 37: Estimated effect sizes of disturbance exposure (in the logit scale). The points represent the estimated effect and the intervals represent the corresponding 95% predictive intervals, for the scenario Temporally-sparse

## Model fit

Figure 38: Model goodness-of-fit showing predicted coral cover versus true values for the prediction-cells. True values corresponds to the mean coral cover at cell-level.

## Cyclone

- Short-term
- Medium-term
- Long-term
- Temporally-sparse

[[1]]

Figure 39: Weighted effects of cyclone exposure per surveyed year for the scenario Short-term

[[1]]

Figure 40: Weighted effects of cyclone exposure per surveyed year for the scenario Medium-term

[[1]]

Figure 41: Weighted effects of cyclone exposure per surveyed year for the scenario Long-term

[[1]]

Figure 42: Weighted effects of cyclone exposure per surveyed year for the scenario Temporally-sparse

## Heat stress

- Short-term
- Medium-term
- Long-term
- Temporally-sparse

[[1]]

Figure 43: Weighted effects of heat stress event per surveyed year for the scenario Short-term

[[1]]

Figure 44: Weighted effects of heat stress event per surveyed year for the scenario Medium-term

[[1]]

Figure 45: Weighted effects of heat stress event per surveyed year for the scenario Long-term

[[1]]

Figure 46: Weighted effects of heat stress event per surveyed year for the scenario Temporally-sparse

## Details on predictive measures

These predictive measures give a single number with low scores representing better performances.

- 95% Coverage Error (CvgErr): The absolute difference between the proportion of times the 95% prediction intervals include the true values and 0.95. It is estimated as follows:

\[
\text{CvgErr}(z, \ell, u) \;=\;
\left| 0.95 \;-\; \frac{1}{n} \sum\_{i=1}^{n} \mathbf{1}\!\left( \ell\_i < z\_i < u\_i \right) \right|
\]

where \(z = \{z\_1, z\_2, \dots, z\_n\}\) are the coral cover observations, \(\ell\) and \(u\) are the lower and upper bounds of the predictive intervals, \(n\) the total number of predictions, and \(\mathbf{1}(\cdot)\) is the indicator function (1 if the condition is true, 0 otherwise).

- Root-Mean-Squared Prediction Error (RMSPE): Indicates how far predictions deviate from true observations, without accounting for uncertainty.

\[
\text{RMSPE} \;=\; \sqrt{ \frac{1}{n} \sum\_{i=1}^{n} (y\_i - \hat{y}\_i)^2 }
\]

where \(y\) and \(\hat{y}\) are the observed and predicted coral cover values, respectively, and \(n\) the total number of observations.

- 95% Interval Score (IS): Balances accuracy and precision by rewarding intervals that include the true value and penalizing those that are too narrow or too wide. It is computed as follow:

\[
\text{IS}\_{95} \;=\; \frac{1}{n} \sum\_{i=1}^{n}
\Bigg[
(u\_i - \ell\_i)
+ \frac{2}{\alpha} (\ell\_i - y\_i)\,\mathbf{1}(y\_i < \ell\_i)
+ \frac{2}{\alpha} (y\_i - u\_i)\,\mathbf{1}(y\_i > u\_i)
\Bigg]
\]

where \(\alpha = 0.05\), \(\ell\) and \(u\) are the lower and upper bounds of the predictive intervals, \(n\) the total number of predictions, and \(y\) are observed coral cover.

- Continuous Ranked Probability Score (CRPS): Assesses the overall quality of the predictive distribution, penalizing inaccuracies, imprecision, and overconfidence.

\[
\text{CRPS}(F, y) \;=\; \sigma \left[
z \left( 2 \Phi(z) - 1 \right) \;+\; 2 \,\phi(z) \;-\; \frac{1}{\sqrt{\pi}}
\right],
\quad z = \frac{y - \mu}{\sigma}
\]

where \(y\) is the observed coral cover values, \(\mu\) and \(\sigma\) are the mean and the standard deviation of the predictive normal distribution, \(\phi(.)\) represented the standard normal probability density function and \(\Phi\) the cumulative distribution function.

## References

British Oceanographic Data Centre (BODC). 2023. “Polygon Dataset of the Extent of Water Bodies from the SeaVoX Salt and Fresh Water Body Gazetteer (V19).” United Kingdom: Available online at https://www.marineregions.org/.

McElreath, Richard. 2018. *Statistical Rethinking: A Bayesian Course with Examples in r and Stan*. Chapman; Hall/CRC.

Zurell, Damaris, Uta Berger, Juliano S Cabral, Florian Jeltsch, Christine N Meynard, Tamara Münkemüller, Nana Nehrbass, et al. 2010. “The Virtual Ecologist Approach: Simulating Data and Observers.” *Oikos* 119 (4): 622–35.
